# Discovery of Kynurenines Containing Oligopeptides as Potent Opioid Receptor Agonists

**DOI:** 10.3390/biom10020284

**Published:** 2020-02-12

**Authors:** Edina Szűcs, Azzurra Stefanucci, Marilisa Pia Dimmito, Ferenc Zádor, Stefano Pieretti, Gokhan Zengin, László Vécsei, Sándor Benyhe, Marianna Nalli, Adriano Mollica

**Affiliations:** 1 Institute of Biochemistry, Biological Research Center, Hungarian Academy of Sciences, Temesvári krt. 62., H-6726 Szeged, Hungary; szucs.edina@brc.hu (E.S.); zador.ferenc@brc.hu (F.Z.); benyhe.sandor@brc.hu (S.B.); 2 Doctoral School of Theoretical Medicine, Faculty of Medicine, University of Szeged, Dómtér 10, H-6720 Szeged, Hungary; 3 Department of Pharmacy, University of Chieti-Pescara “G. d’Annunzio”, Via dei Vestini 31, 66100 Chieti, Italy; marilisa.dimmito@unich.it (M.P.D.); a.mollica@unich.it (A.M.); 4 National Center for Drug Research and Evaluation, Istituto Superiore di Sanità, Viale Regina Elena 299, 00161 Rome, Italy; stefano.pieretti@iss.it; 5 Department of Biology, Science Faculty, Selcuk University, 42250 Konya, Turkey; gokhanzengin@selcuk.edu.tr; 6 MTA-SZTE Neuroscience Research Group, Department of Neurology, Interdisciplinary Excellence Centre, Faculty of Medicine, University of Szeged, H-6725 Szeged, Hungary; vecsei.laszlo@med.u-szeged.hu; 7 Laboratory affiliated with the Institute Pasteur Italy-Cenci Bolognetti Foundation, Department of Drug Chemistry and Technologies, Sapienza University of Rome, Piazzale Aldo Moro 5, I-00185 Roma, Italy; marianna.nalli@uniroma1.it

**Keywords:** peptides, kynurenines, binding affinity, μ-opioid receptor, pharmacophore, G-protein activation

## Abstract

Kynurenine (kyn) and kynurenic acid (kyna) are well-defined metabolites of tryptophan catabolism collectively known as “kynurenines”, which exert regulatory functions in host-microbiome signaling, immune cell response, and neuronal excitability. Kynurenine containing peptides endowed with opioid receptor activity have been isolated from natural organisms; thus, in this work, novel opioid peptide analogs incorporating L-kynurenine (L-kyn) and kynurenic acid (kyna) in place of native amino acids have been designed and synthesized with the aim to investigate the biological effect of these modifications. The kyna-containing peptide (**KA1**) binds selectively the μ-opioid receptor with a Ki = 1.08 ± 0.26 (selectivity ratio μ/δ/κ = 1:514:10,000), while the L-kyn-containing peptide (**K6**) shows a mixed binding affinity for μ, δ, and κ-opioid receptors, with efficacy and potency (E_max_ = 209.7 + 3.4%; LogEC_50_ = −5.984 + 0.054) higher than those of the reference compound DAMGO. This novel oligopeptide exhibits a strong antinociceptive effect after i.c.v. and s.c. administrations in in vivo tests, according to good stability in human plasma (t_1/2_ = 47 min).

## 1. Introduction

**Figure 1:**
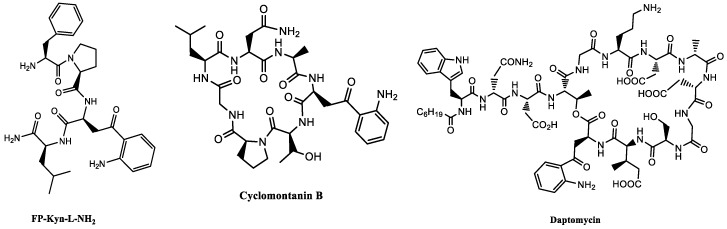
Natural bioactive compounds containing kyn residue.

The kynurenine pathway (KP) is an essential part of the tryptophan metabolism in mammalian tissues, where it is responsible for the formation of two principal metabolites, namely, L-kynurenine (kyn) and kynurenic acid (kyna). Kyn can arise in peptides and proteins by post/translational modifications or direct oxidation of tryptophan. It is present in lens crystallins, human Cu^2+^/Zn^2+^ dismutase, milk proteins, actin oxidized in vivo, and several bioactive compounds produced by bacteria and marine organisms [1]. Daptomycin is a cyclic kyn-containing lipodepsipeptide approved by the Food and Drug Administration (FDA), isolated from *Streptomyces roseoporus* used in the treatment of Gram-positive pathogen skin infections [2]. Cyclomontanin B isolated from *Annona montana* exhibits promising anti-inflammatory activity [3]. The kyn-containing peptide FP-Kyn-L-NH_2_ is the minor component of Australian red tree frog skin *Litoria rubella* collected in central Australia, endowed with opioid activity at 10^−7^ M (Figure 1) [4].

The occurrence of kyn in natural products suggests a possible specificity towards their biological targets. The enzymes of the human kynurenine pathway are expressed in different tissues and cell types throughout the body [1]. In humans, the majority of kyn is excreted by urine; thus, its bioavailability increases according to the tryptophan flux downstream of the KP [5]. Kyn is able to penetrate the central nervous system (CNS) by transport across the blood–brain barrier (BBB), but it is also produced locally [6].

Kyna has been originally discovered in canine urine, but a huge amount has been measured in the gut, bile, human saliva, synovial and amniotic fluid; it has also been detected in food products such as broccoli, some potatoes, and honeybee products [1]. Kyna possesses an antagonistic effect on the N-methyl-D-aspartate (NMDA) receptor and other glutamate receptors such as AMPA and kainate receptors [7,8]. Kyna is also found to have an agonistic effect on the G protein coupled receptor GPR35 [9,10], which can be found in various tissues and organs such as gastrointestinal tract, liver, immune system, central nervous system, and cardiovascular system [11]. NMDA receptors are essential for the control of the glutamatergic work at the CNS; in contrast to the kainate and AMPA receptors, the NMDA mediates the influx of Ca^2+^ ions into neurons, playing an important role in synaptic plasticity, memory, and learning [7,8]. Overactivation of NMDA receptors can lead to excitotoxicity, severe cell damage, and apoptosis of neurons, which are strongly related to neurodegenerative and CNS disorders such as depression, stroke, ischemia, and neuropathic pain [10,11,12]. Different therapeutic approaches based on thekynurenine pathway have been postulated to circumvent this problem, such as the use of kynurenic acid prodrugs or analogs able to penetrate more readily than the parent compounds or the involvement of ascorbate conjugation to promote the interaction of kyna with SVCT2 transport protein [13,14,15]. Intracisternal kyna attenuates formalin-induced nociception in animals together with antagonist activity at the glycine binding site of NMDA, which is associated with analgesic properties in rats [16]. At the peripheral sites, kyna decreases the nociceptive behavior in the tail flick and hot plate tests [16]. Administration of L-kyn and probenecid together with kyna analogs inhibits NMDA receptors in animal models of trigeminal activation and sensitization [17]. Noteworthy, kyna and its analogs are able to act on second-order neurons, decreasing mechanical allodynia and pain sensitivity in different animal pain models [18]. Considering the presence of kyn residue in natural peptide sequences and the important role exerted by both kynurenines at the CNS [19,20,21], we plan to investigate the biological consequences of the insertion of these residues in opioid pharmacophoric sequences. Kyn could be used in place of phenylalanine, considering its aromatic side chain, whereas kyna could be used as *C*-terminus to mimic an additional aromatic residue. In this preliminary work, we performed the synthesis and biological screening of six novel kynurenines containing peptides, aiming to investigate the modifications imposed by the presence of kyn and kyna on the biological properties of known endogenous and synthetic opioid peptides in vivo and in vitro. Peptide **KA1** retains the DAMGO primary sequence, but the OH terminal group is esterified by kynurenic acid. Peptides **K2** and **K3** are EM-2 analogs in which the Phe residues in positions 3 and 4 have been replaced with kyn and kyn *C*-terminal amides, respectively. Peptides **K4–K6** are enkephalin-like peptides containing kyn in position 5, bearing as *C*-terminal the methyl ester, acid, and amide group, respectively. The novel chemical entities were prepared following solution phase peptide synthesis and were obtained as TFA salts in good overall yields and excellent purities.

## 2. Materials and Methods

### 2.1. Chemistry

All reagents and solvents were acquired from Sigma-Aldrich (Milano, Italy). Solution phase peptide synthesis was applied to prepare the final products **KA1, K2–K6** as TFA salts, following the procedures reported below. Boc-protected intermediates were purified by silica gel column chromatography where necessary, or by trituration in Et_2_O. Final products **KA1**, **K2–K6** were purified by RP–HPLC on a Waters XBridge BEH130 (C18 5.0 μm, 250 × 10 mm column; flow rate of 7 mL/min; Waters Binary pump 1525; eluent: linear gradient of H_2_O/ACN 0.1% TFA, ranging from 5% to 95% ACN in 32 min). The purity of the *N*^α^-Boc-protected products was confirmed by NMR analysis on a Varian Mercury 300 MHz. The purity of all final compounds was assessed by NMR analysis, ESI–LRMS, and by analytical RP–HPLC (C18-bonded 4.6 × 150 mm; flow rate of 1 mL/min; eluent: gradient of H_2_O/ACN 0.1% TFA, ranging from 5% to 95% ACN in 26 min, recorded at 254, 275, and 213 nm and was found to be ≥95%). The mass spectrometry (MS) equipment was composed as follows: LCQ Thermo Finnigan ion trap mass spectrometer (San Jose, CA, USA) with an electrospray ionization (ESI) source; capillary temperature: 300 °C; spray voltage: 4.00 kV; nitrogen (N_2_) as the sheath and auxiliary gas.

### 2.2. General Procedures

#### 2.2.1. Formation of Ethanolamine-Kynurenic Acid Ester

Kynurenic acid (2 mmol, 1 equiv.) was dissolved in DMF (5 mL) stirring in agitation at 0 °C, then a mixture of Boc-*N*-aminoethanol (2 mmol, 1 equiv.) and DMAP (0.6 mmol, 0.3 equiv.) in DMF (3 mL) was transferred in the round bottom flask. After 10 min, EDC.HCl (2.2 mmol, 1.1 equiv.) was added to the reaction mixture in agitation at 0 °C for 10 min, then at r.t. overnight. The solvent was removed in a rotary evaporator and the oily residue was taken up with EtOAc and washed with 5% citric acid solution (3 times), NaHCO_3_ s.s. (3 times), and NaCl s.s. (3 times). Organic phases were collected and dried on Na_2_SO_4_ anhydrous, filtered and dried in rotavapor and high vacuum to give a yellow oily product. The crude product was triturated with Et_2_O (2 times), the aqueous layer filtered up, and the white solid product dried in a rotary evaporator and high vacuum.

#### 2.2.2. Coupling Reaction

Boc-protected compound (1.1 equiv.) was dissolved in DMF (5 mL) in an iced-cooled bottom flask, then EDC.HCl (1.1 equiv.) and HOBt hydrate (1.1 equiv.) were added stirring for 10 min. A solution of *N*-terminal free intermediate (1 equiv.) and DIPEA (3.3 equiv.) in DMF (5 mL) was transferred in the ice-cooled bottom flask at 0 °C and allowed to react at r.t. overnight. The solvent was removed in a rotary evaporator; the oily residue was taken up with EtOAc and washed with 5% citric acid solution (3 times), NaHCO_3_ s.s. (3 times), and NaCl s.s. (3 times). Organic phases were collected and dried on Na_2_SO_4_ anhydrous, filtered and dried in a rotavapor and high vacuum to give a yellow oily product. The crude product was triturated with Et_2_O (2 times), the aqueous layer filtered up and the white solid product dried in a rotary evaporator and high vacuum.

#### 2.2.3. Amidation

The Boc-protected or free *N*-terminal compound (1 equiv.) was dissolved in THF (7 mL) stirring at −15 °C, then NMM (2.5 equiv.) and *i*BCF (2.1 equiv.) were added to the solution allowing to react for 30 min. Then NH_4_OH aq. solution (0.21 mL) was added to the reaction mixture at −15 °C for 30 min. The reaction mixture was allowed to react at r.t. for 2 h. The solvent was removed in rotavapor and the solid residue was dissolved in EtOAc washing with 5% citric acid solution (3 times), NaHCO_3_ s.s. (3 times), and NaCl s.s. (3 times). Organic phases were collected and dried on Na_2_SO_4_ anhydrous, filtered and dried in a rotavapor and high vacuum to give a yellow oily product. The crude product was triturated with Et_2_O (2 times), the aqueous layer filtered up, and the product dried in a rotary evaporator and high vacuum to give a white solid product.

#### 2.2.4. Saponification

Boc-protected compound (0.1 mmol, 1 equiv.) was dissolved in THF (5 mL) stirring at r.t. then NaOH 1M (4 equiv.) was added dropwise, allowing it to react for 3 h. The solvent was removed in a rotavapor; the oily residue was taken up with water and washed with Et_2_O (2 times). The aqueous solution was acidified with HCl 1 M until complete precipitation of the solid residue, which was extracted with EtOAc 3 times. The organic layers were collected, dried on Na_2_SO_4_ anhydrous, filtered and dried in a rotavapor and high vacuum to give a white solid product.

### 2.3. Synthesis and Characterization

Description of the reaction procedures [22,23,24] and compounds characterization are reported in detail in the Supplementary Materials.

### 2.4. In Vitro Biological Assays

#### 2.4.1. Chemicals

Tris-HCl, EGTA, NaCl, MgCl_2_·6H_2_O, GDP, the GTP analog GTPγS, and the L-tryptophan metabolite kynurenic acid were from Sigma-Aldrich (Budapest, Hungary); Tyr-D-Ala-Gly-(NMe)Phe-Gly-ol (DAMGO) was purchased from Bachem Holding AG (Bubendorf, Switzerland); endomorphin-2 (EM-2) was kindly provided by MTA-ELTE Research Group of Peptide Chemistry (Budapest, Hungary); Ile^5,6^-deltorphine II (Ile^5,6^Delt II) was purchased from Isotope Laboratory of BRC (Szeged, Hungary); and the highly selective KOR agonist diphenethylamine derivative, HS665 [25], was offered by Dr. Helmut Schmidhammer (University of Innsbruck, Austria). Naloxone was provided by Endo Laboratories DuPont de Nemours (Wilmington, DE, USA). The non-competitive NMDA antagonist, (+)-MK 801 maleate (MK-801) and L-kynurenine (L-kyn) were obtained from Tocris Bioscience (Bristol, UK). A solution of each ligand in water was stored in 1 mM stock solution at −20 °C. The radiolabelled GTP analog, [^35^S]GTPγS (specific activity: 1000 Ci/mmol), was acquired from Hartmann Analytic (Braunschweig, Germany). [^3^H]DAMGO [26] (specific activity: 38.8 Ci/mmol), [^3^H]Ile^5,6^Delt II (specific activity: 19.6 Ci/mmol), and [^3^H]HS665 [27] (specific activity: 13.1 Ci/mmol) were radiolabelled in the Isotope Laboratory of BRC (Szeged, Hungary). [^3^H]MK-801 [28] (specific activity: 30 Ci/mmol) was purchased from PerkinElmer (Boston, MA, USA) and the UltimaGold^TM^ MV aqueous scintillation cocktail was from PerkinElmer (Boston, MA, USA).

#### 2.4.2. Animal

Male and female Wistar rats and guinea pigs were used for membrane preparations. The animals were guarded in a temperature-controlled room, ranging from 21 to 24 °C, under a 12:12 light and dark cycle with water and food ad libitum. All housing and experiments were conducted in accordance with the European Communities Council Directives (86/606/ECC) and the Hungarian Act for the Protection of Animals in Research (XXVIII.tv. 32.§). The total number of animals, as well as their suffering, was minimized whenever possible.

#### 2.4.3. Preparation of Brain Samples for Binding Assays

Rats and guinea pigs were decapitated, and their brains were quickly removed. The brains were used for membrane preparation following the procedure reported by Benyhe [29] for binding and [^35^S]GTPγS binding experiments, in agreement with the protocol of Zádor et al. [30].

Homogenization of brains was performed in 30 volumes (*v/w*) of ice-cold 50 mM Tris-HCl pH 7.4 buffer with a Teflon-glass Braun homogenizer at 1500 rpm. The centrifuge was settled at 18,000 rpm for 20 min at 4 °C, the resulting supernatant discarded, and the pellet taken up in the original volume of Tris-HCl buffer. Incubation of homogenate at 37 °C for 30 min was performed in a shaking water-bath. Five volumes of 50 mM Tris-HCl pH 7.4 buffer were used to suspend the final pellet at −80 °C. For the [^35^S]GTPγS binding experiments, the brains were homogenized with a Dounce in 5 volumes (*v/w*) of ice-cold TEM (Tris-HCl, EGTA, MgCl_2_) at −80 °C. The protein content of the membrane preparation was determined by the method of Bradford, BSA being used as a standard [31].

#### 2.4.4. Receptor Binding Assays

##### Functional [^35^S]GTPγS Binding Experiments

The functional [^35^S]GTPγS binding experiments were performed as previously described [32,33]. Briefly the membrane homogenates were incubated at 30 °C for 60 min in Tris-EGTA buffer (pH 7.4) composed of 50 mM Tris-HCl, 1 mM EGTA, 3 mM MgCl_2_, 100 mM NaCl, containing 20 MBq/0.05 cm^3^ [^35^S]GTPγS (0.05 nM) and increasing concentrations (10^−10^ to 10^−5^ M) of ligands. The experiments were performed in the presence of excess GDP (30 μM) in a final volume of 1 mL. Total binding was measured in the absence of test compounds, non-specific binding was determined in the presence of 10 μM unlabeled GTPγS. The reaction was terminated by rapid filtration under vacuum and washed three times with 5 mL ice-cold 50 mM Tris-HCl (pH 7.4) buffer through Whatman GF/B glass fibers. The radioactivity of the filters was detected in an UltimaGold^TM^ MV aqueous scintillation cocktail with a Packard Tricarb 2300TR liquid scintillation counter.

##### Binding Experiments

In MOR, DOR, and KOR displacement, aliquots of frozen rat and guinea pig brain membrane homogenates were thawed and suspended in 50 mM Tris-HCl buffer (pH 7.4); in NMDA displacement, the Tris-HCl buffer (pH 7.4) contained 100 µM glycine and 100 µM L-glutamic acid. Samples were incubated in the presence of the unlabeled ligands in increasing concentrations (10^−10^ to 10^−5^ M) for at 35 °C for 45 min with [^3^H]DAMGO, for at 35 °C for 45 min with [^3^H]Ile^5,6^Delt II, at 25 °C for 30 min with [^3^H]HS665, and at 25 °C for 120 min with [^3^H]MK-801. The non-specific and total binding was determined in the presence and absence of 10 μM unlabelled naloxone (MOR and DOR), HS665 (KOR), and MK-801 (NMDA). Radioactivity of the filter disks was measured, as written above.

##### Data Analysis

Experimental data are presented as means ± S.E.M. Sigmoid dose-response curves were fitted with GraphPad Prism 5.0 (GraphPad Prism Software Inc., San Diego, CA, USA). An unpaired *t*-test with two-tailed *p* value was performed to determine the significance level. In the competition binding assays, the ‘One site competition’ fitting was used to establish the equilibrium binding affinity (K_i_ value).

### 2.5. In Vivo Tests

#### 2.5.1. Animals

In our experiments, we used CD-1 male mice (Harlan, Italy, 25–30 g) maintained in colony, housed in cages (7 mice per cage) under standard light/dark cycle (from 7:00 a.m. to 7:00 p.m.), temperature (21 ± 1 °C) and relative humidity (60% ± 10%) for at least 1 week. Food and water were available ad libitum. The Service for Biotechnology and Animal Welfare of the Istituto Superiore di Sanità and the Italian Ministry of Health authorized the experimental protocol according to Legislative Decree 26/14.

#### 2.5.2. Treatment Procedure

DMSO was purchased from Merck (Rome, Italy). Peptides solutions were freshly prepared using saline containing 0.9% NaCl and DMSO in the ratio DMSO/saline 1:5 *v/v* every experimental day. These solutions were injected at a volume of 10 μL/mouse for intracerebroventricular (i.c.v.) administrations or at a volume of 20 μL/mouse for subcutaneous administrations.

#### 2.5.3. Surgery for i.c.v. Injections

For i.c.v. injections, mice were lightly anesthetized with isoflurane, and an incision was made in the scalp, and the bregma was located. Injections were performed using a 10 μL Hamilton microsyringe equipped with a 26-gauge needle, 2 mm caudal and 2 mm lateral from the bregma at a depth of 3 mm.

#### 2.5.4. Tail Flick Test

The tail flick latency was obtained using a commercial unit (Ugo Basile, Gemonio, Italy), consisting of an infrared radiant light source (100 W, 15 V bulb) focused onto a photocell utilizing an aluminum parabolic mirror. During the trials, the mice were gently hand-restrained using leather gloves. Radiant heat was focused 3–4 cm from the tip of the tail, and the latency (s) of the tail withdrawal to the thermal stimulus was recorded. The measurement was interrupted if the latency exceeded the cut off time (15 s at 15 V). The baseline latency was calculated as mean of three readings recorded before testing at intervals of 15 min and the time course of latency determined at 15, 30, 45, 60, 90, and 120 min after treatment. Data were expressed as time course of the maximum percentage effect (%MPE) = (post-drug latency − baseline latency)/(cut-off time − baseline latency) × 100.

#### 2.5.5. Formalin Test

In the formalin test, the injection of a dilute solution of formalin (1%, 20 μL/paw) into the dorsal surface of the mouse hind paw evoked biphasic nociceptive behavioral responses, such as licking, biting the injected paw, or both, occurring from 0 to 10 min after formalin injection (the early phase) and a prolonged phase, occurring from 15 to 40 min (the late phase). Before the test, mice were individually placed in a Plexiglas observation cage (30 × 14 × 12 cm) for one hour, to acclimatize to the testing environment. The total time the animal spent licking or biting its paw during the early and late phase of formalin-induced nociception was recorded.

#### 2.5.6. Data Analysis and Statistics

Experimental data were expressed as mean ± s.e.m. In the tail flick test, significant differences among the groups were evaluated with two-way ANOVA followed by Sidak’s multiple comparisons test. Formalin test data were analyzed by using one-way ANOVA, followed by Holm–Sidak’s multiple comparisons test. GraphPad Prism 6.03 software was used for all the analyses. Statistical significance was set at *p* < 0.05. The data and statistical analysis comply with the recommendations on experimental design and analysis in pharmacology.

### 2.6. Stability in Human Plasma Sample Preparation

Five microliters of **K6** (500 μg in 250 μL of water) were added to 45 μL of fresh human plasma, then incubated at 37 ± 1 °C. Prepared samples were removed at several designated time points and incubation was stopped by adding an equal volume of the sequencing mixture (5% aqueous ZnSO_4_ solution, MeOH, and ACN; 5:3:2), which precipitated proteins, to achieve a final concentration of 100 μg/mL. The mixture was vortexed and centrifuged at 12,000× *g* for 5 min, then 20 μL of clear supernatant was directly injected into the HPLC system (Waters model 600 solvent pump and 2996 photodiode array detector, with XBridge BEH 130 C18, 4.6 × 250 mm, 5 μm). The samples were tested in three independents experiments (n = 3) and reported values represent the mean ± standard error (SEM). Data were analysed using simple regression analysis (significant deviation from zero: F_1,13_ = 171.9, *p* < 0.0001; Y = −0.9705 × X + 95.98).

## 3. Results and Discussion

### 3.1. Chemistry

Simple modifications of kyna scaffold through the insertion of aromatic substituents [34], *C*-terminal derivatization as methyl ester [35], or amide bearing a water-soluble side chain [36] have been abundantly documented in the literature, rather than the insertion of kyna into a peptide sequence. On the contrary, different papers highlight the scarce propensity of the kyn carboxylic group to react with *N*-terminus free amino acids involved in the coupling reaction, which renders this unusual amino acid difficult to manage as a building block for peptide synthesis [22,23].

Taking this in mind, we focused our attention on the functionalization of kyna via a variant of Steglich esterification with the previously prepared Boc-protected 2-aminoethanol [23] so as to obtain intermediate **2** in 60% yield after purification by column chromatography (see General Procedure) [24]. Compound **2** was deprotected with a mixture of TFA:DCM = 1:1 at r.t. for 1 h and the so obtained intermediate was coupled with Boc-*N*(Me)Phe-OH, following the standard procedure for coupling reaction [36].

**Scheme 1:**
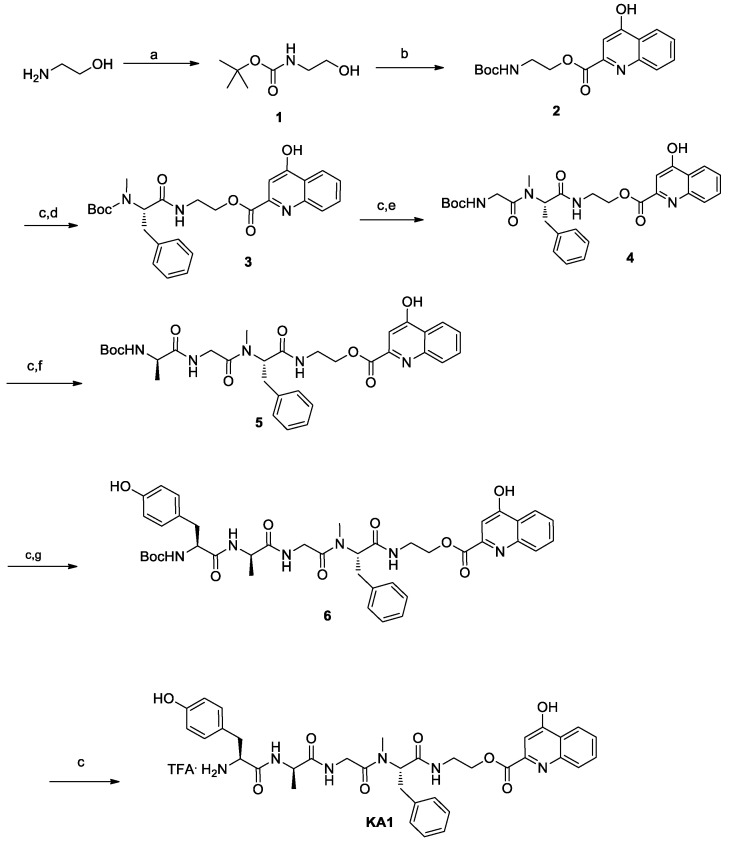
Synthesis of peptide **KA1**. Reagents and conditions: (**a**) Boc_2_O, NaOH 1 M, THF, 10 min at 0 °C, then 16 h at r.t. quantitative; (**b**) kynurenic acid, EDC·HCl, DMAP, DMF, 10 min at 0 °C, then 36 h at r.t., 60% yield after silica gel column chromatography; (**c**) TFA/DCM 1:1 1 h at r.t. quantitative; (**d**) Boc-*N*-Me-Phe-OH, EDC·HCl, HOBt, DIPEA, DMF, 10 min at 0 °C, then 16 h at r.t., 75% yield after reaction work-up; (**e**) Boc-Gly-OH, EDC·HCl, HOBt, DIPEA, DMF, 10 min at 0 °C, then 16 h at r.t., 70% yield after reaction work-up; (**f**) Boc-DAla-OH, EDC·HCl, HOBt, DIPEA, DMF, 10 min at 0 °C, then 16 h at r.t., 80% yield after silica gel column chromatography; (**g**) Boc-Tyr-OH, EDC·HCl, HOBt, DIPEA, DMF, 10 min at 0 °C, then 16 h at r.t., 66% yield from **5**, after silica gel column chromatography.

Peptide elongation/deprotection steps were repeated to reach the complete Boc-protected peptide **6** in 66% yield, starting from intermediate compound **5,** after silica gel column chromatography (Scheme 1). The final peptide **KA1** has been obtained in 71% yield after RP–HPLC purification. The purity of the sample was assessed by analytical RP–HPLC recorded at 254 nm and was found to be ≥95%.

**Scheme 2:**
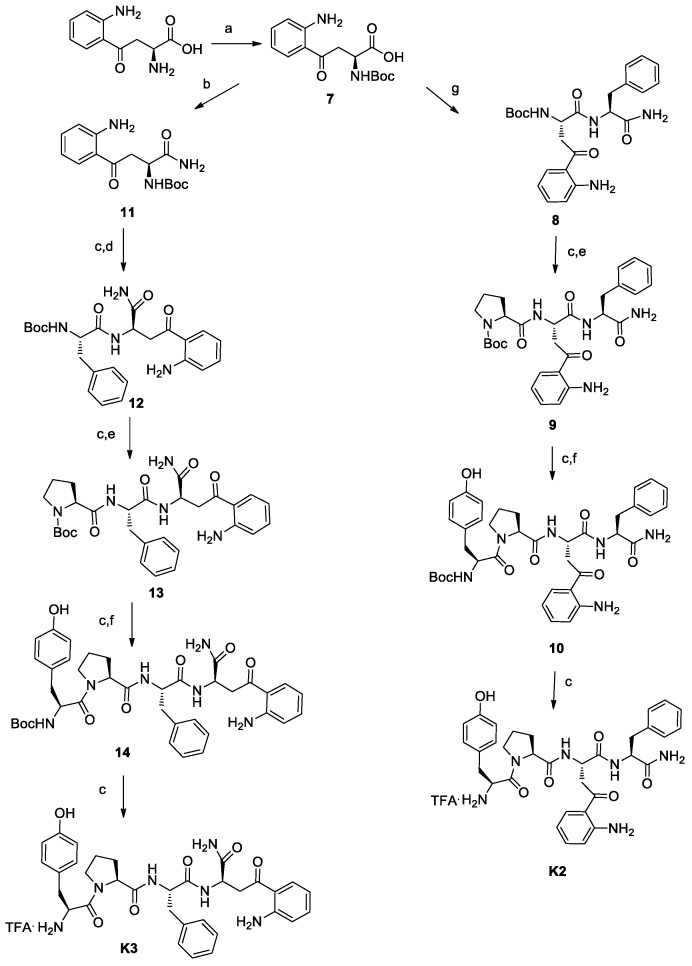
Synthesis of peptides **K2, K3**. Reagents and conditions: (**a**) Boc_2_O, NaHCO_3_, NaOH 1 M, dioxane/H_2_O (2:1), 10 min at 0 °C, then 30 min at r.t. quantitative; (**b**) *i*BCF, NMM, NH_4_OH, THF, 30 min at −10 °C, then 16 h at r.t., 71% yield after trituration; (**c**) TFA/DCM 1:1, 1 h at r.t. quantitative; (**d**) Boc-Phe-OH, EDC^.^HCl, HOBt, DIPEA, DMF, 10 min at 0 °C, then 16 h at r.t. quantitative; (**e**) Boc-Pro-OH, EDC·HCl, HOBt, DIPEA, DMF, 10 min at 0 °C, 16 h at r.t., 72% yield for **13** after trituration, 81% yield for **9**; (**f**) Boc-Tyr-OH, EDC·HCl, HOBt, DIPEA, DMF, 10 min at 0 °C, 16 h at r.t., 95% yield for **14** after trituration, 73% yield for **10**; (**g**) H-Phe-NH_2_, EDC·HCl, HOBt, DIPEA, DMF, 10 min at 0 °C, then 16 h at r.t., 61% yield from **7** after trituration.

Then L-kyn was converted in its Boc-derivative **7** following the procedure reported by Tsentalovich et al. [23] to prepare the EM-2 analogs **K2** and **K3** (Scheme 2). Firstly intermediate **7** was coupled with H-Phe-NH_2_, previously prepared following the general procedure of amidation, to obtain intermediate **8** in 61% yield. The Boc-protecting group was removed from compound **8**, and the so obtained TFA salt was coupled with Boc-Pro-OH to give intermediate **9** in good yield (81%). Repeated steps of coupling/purification/deprotection afforded the final compound **K2** in 72% yield from **10**, and excellent purity after RP–HPLC purification of the crude product. Conversely, Boc-Kynurenine **7** was transformed in the amide Boc-derivative **11** in a 71% yield. Then it was deprotected with a mixture of TFA:DCM = 1:1 at r.t. for 1 h and the so obtained TFA salt was coupled with BocPhe-OH to afford intermediate **12** quantitatively. Then repeated steps of coupling/purification/deprotection were performed to reach peptide **K3** in high yield (83% from **14**) and excellent purity after RP–HPLC purification of the crude compound.

**Scheme 3:**
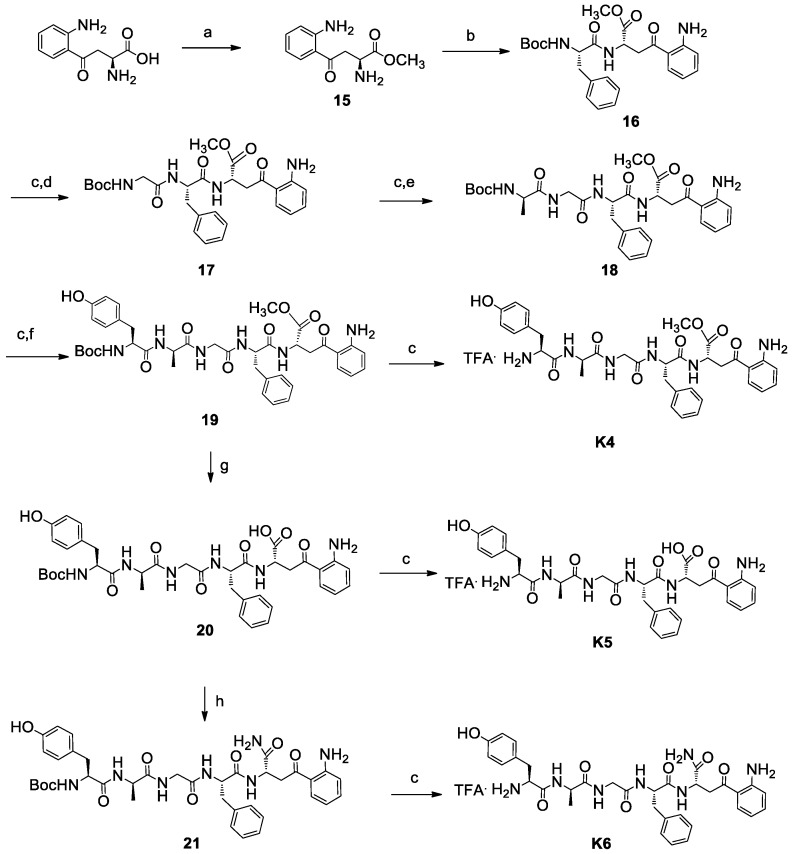
Synthesis of linear peptides **K4–K6**. Reagents and conditions: (**a**) SOCl_2_, MeOH, 30 min at 0 °C, 16 h at r.t., quantitative; (**b**) Boc-Phe-OH, EDC·HCl, HOBt, DIPEA, DMF, 10 min at 0 °C, then 16 h at r.t., 60% after silica gel column chromatography; (**c**) TFA/DCM 1:1, 1 h at r.t. quantitative; (**d**) Boc-Gly-OH, EDC·HCl, HOBt, DIPEA, DMF, 10 min at 0 °C, 16 h at r.t., quantitative; (**e**) Boc-DAla-OH, EDC·HCl, HOBt, DIPEA, DMF, 10 min at 0 °C, 16 h at r.t., 84% yield after silica gel column chromatography; (**f**) Boc-Tyr-OH, EDC·HCl, HOBt, DIPEA, DMF, 10 min at 0 °C, 16 h at r.t., 72% yield after trituration; (**g**) NaOH 1 M, THF, 3 h at r.t., quantitative; (**h**) *i*BCF, NMM, NH_4_OH (aq), THF, 30 min at −15 °C, 2 h at r.t., 52% yield after trituration.

Finally, linear peptides **K4–K6** have been synthesized as methyl ester, acid, and amide derivatives, respectively, starting from L-kyn methyl ester **15** (Scheme 3), prepared following the procedure described in the literature [24,36]. Repeated steps of deprotection/coupling reaction were performed to reach intermediate **19** in high yield after trituration in Et_2_O. Boc group removal of intermediate **19** gave **K4** in good yield and excellent purity after RP–HPLC purification. Saponification of intermediate compound **19** afforded **20,** following the general procedure; the conversion of the latter in the amide **21** was performed as previously described by Stefanucci et al. [24]. Boc group removal of the linear peptides **20** and **21** afforded products **K5** and **K6** in good yields (72% and 52%, respectively), and excellent purity after RP–HPLC purification (98% and 96%, respectively).

### 3.2. In Vitro Studies

#### 3.2.1. Binding Assays

Kyna and its analog (KYNA1) previously reported by Zádor et al. did not directly bind μ, δ, κ-receptors in vitro [37]. However, after chronic and acute administration, they altered opioid receptor function in vivo and in vitro through the NMDA receptor co-localized in the cortex and striatum of mice and rats, though the interaction of opioid receptors and NMDA have been deeply discussed in the literature [37,38]. Kyna is able to bind to the NMDA receptor at micromolar affinity [38]. To test if our novel peptides are able to target both of these systems, they were examined in a receptor binding radioassay using highly specific tritium-labelled primary ligands for opioid and NMDA receptor binding sites. [^3^H]DAMGO, [^3^H]Ile^5,6^Delt II, and [^3^H]MK-801 equilibrium competition (displacement) studies were conducted in rat brain homogenates, while κ-opioid receptor tests were performed with [^3^H]HS665 in guinea pig brain homogenates. The novel ligands showed similar equilibrium inhibitory affinities (K_i_ value) in the μ-opioid system as DAMGO except **K3** (Table 1, Figure S1A). In the δ-opioid system, the ligands showed lower binding affinity (higher Ki) compared to the selective δ-opioid receptor selective agonist Ile^5,6^Delt II (Figure S1B). In the κ-opioid receptor system, the compounds showed higher Ki values than that of the selective κ-opioid agonist HS665 (Figure S1C). In the NMDA receptor binding assays, the peptides did not produce any competing activity (Figure S1D). Peptide **KA1** possesses the best binding affinity, with a Ki value very close to that of the reference compound DAMGO (1.08 ± 0.26 nM vs. 0.90 ± 0.28 nM), suggesting that the insertion of kyna into the DAMGO sequence does not impair its binding potency at the µ-opioid receptor. The peptide **K6,** presenting the enkephalin-like structure linked to L-kyn *C*-terminal amide, is able to bind all three opioid receptors with significant affinity, showing a moderate preference for the µ-opioid receptor (affinity ratio 1:18:70 for μ, δ, k, respectively), whereas compounds **K4** and **K5** are able to bind only μ- and δ-opioid receptors. It is reasonable to believe that the *C*-terminal amide derivatization in peptide **K6** confers the ability to bind to the κ-opioid receptor. Concerning the endomorphin-2 (EM-2) analogs **K2** and **K3**, the replacement of Phe^3^ with L-kyn improves the binding affinity and selectivity of **K2** for μ-opioid receptors with respect to the standard compound EM-2, with a weak affinity for κ-opioid receptors, while the incorporation of L-Kyn amide in position 4 causes the loss of selectivity for MOR in favor of a modest binding affinity for μ- and δ-opioid receptors. Peptide **K2** shows a Ki value two-folds lower than that of EM-2 on the µ-opioid receptor, which let us suppose the positive influence of L-Kyn in position 3 on **K2** binding ability.

#### 3.2.2. Binding-Protein Activation Assays

The effect of kyn or kyna combined peptides on G-protein activation was investigated in functional [^35^S]GTPγS binding assays in rat and guinea pig brain membranes. All ligands produced dose-dependent stimulations described by sigmoid curves (Figure S2). **K6** showed higher efficacy (E_max_) than DAMGO (Table 2). Moreover, 10 µM cyprodime and 10 µM naltrindole, which are selective MOR and DOR antagonists, respectively [40,41], significantly reversed the agonist effects of the ligands in rat brain membrane homogenates (Figure S3A). In guinea pig brain membrane homogenates, the ligands did not activate G-protein except **K4** and **K6** (Figure S3B). Additionally, 10 µM norbinaltorphine decreased significantly the agonist effect of **K4** but did not change the effect of **K6** (Table 3). Altogether, these data reveal that peptide **KA1** acts as a selective μ-opioid agonist, being able to decrease the GTPγS binding percentage under the basal level in the presence of 10 μM cyprodime. Peptides **K2** and **K3** possess a mixed μ/δ agonist activity profile, while peptides **K4–K6** show a modest mixed μ/δ-opioid receptor agonism. Probably peptide **K6** is the strongest mixed μ/δ/κ opioid agonist due to its ability to bind protein G with an efficacy value over the basal level in presence of each selective opioid antagonist at 10 μM.

### 3.3. In Vivo Studies

**Figure 2:**
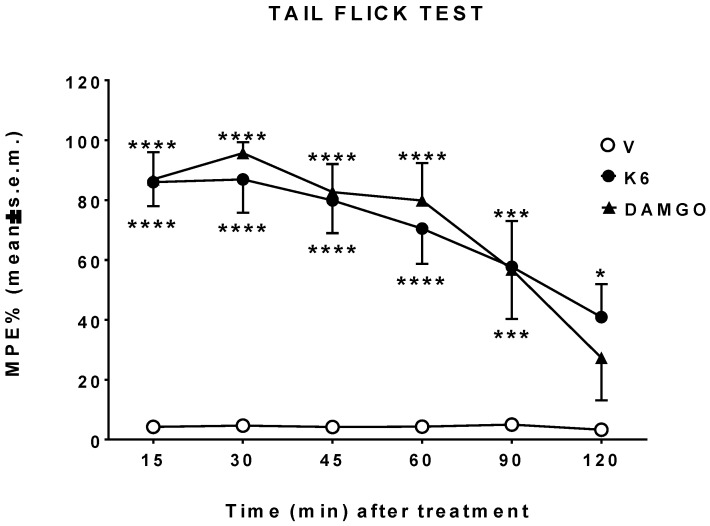
The effect of **K6** and DAMGO in the tail flick test. Compounds were administered in the left cerebral ventricle at the dose of 10 μg/10 μL, and the time to respond to thermal stimuli measured from 15 to 120 min. V is for vehicle-treated animals. Statistical analysis: two-way ANOVA followed by Sidak’s multiple comparisons test. * is for *p* < 0.05, *** is for *p* < 0.001, and **** is for *p* < 0.0001 vs. V. n = 7.

Data obtained from binding experiments indicate **K6** as the best of the series; thus, tail flick and formalin tests were performed in order to evaluate the effects of this novel peptide in two different pain animal models. The tail flick test measures the time in which the animal withdraws the tail from a thermal nociceptive stimulus; this time is increased by analgesics. Compounds were centrally injected, and the response measured from 15 to 120 min after the administration. Compounds **K6** and DAMGO greatly increase the time of response to thermal nociceptive stimuli and both effects were in the same order to magnitude (Figure 2).

**Figure 3:**
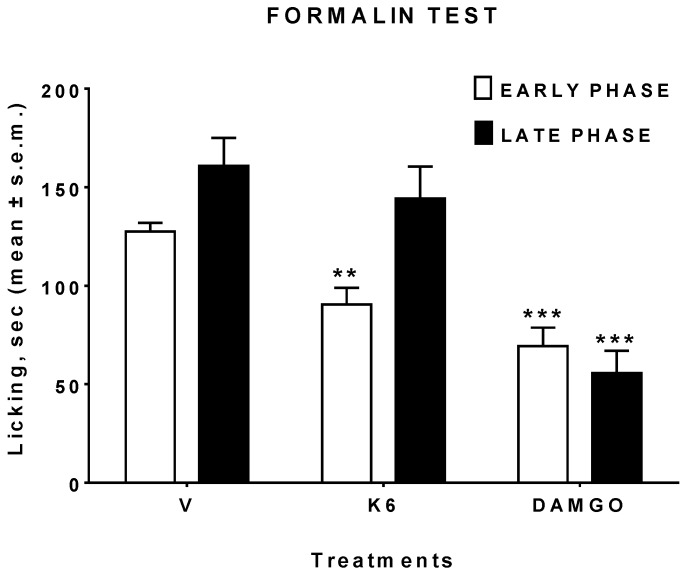
The effect of **K6** and DAMGO in the formalin test. Compounds were administered subcutaneously (s.c.) in the dorsal hind paw of mice at the dose of 100 μg/20 μL, 15 min before a s.c. injection of dilute formalin solution (1% in saline, 20 μL/paw). Early phase represents the formalin-induced nociceptive behavior recorded from 0 to 10 min after formalin injection; late phase is for the formalin-induced nociceptive behavior recorded from 15 to 40 min after formalin injection. V is for vehicle-treated animals. Statistical analysis: one-way ANOVA followed by Holm–Sidak’s multiple comparisons test. ** is for *p* < 0.01 and *** is for *p* < 0.001 vs. V. n = 7.

Since it was reported that some opioid peptides such as DAMGO are also peripheral acting [42], we performed a formalin test administering **K6** and DAMGO subcutaneously in mice paws. The formalin test measures the behavioral response to chemical nociceptive stimuli evoked by a formalin diluted solution injected in the mice paw. From 0 to 10 min after the injection, an early phase response occurs with direct stimulation of peripheral nociceptors, while a response to inflammatory pain appears from 15 to 40 min as a late prolonged phase. Compound **K6** was able to reduce the nociceptive response to formalin in the early phase, whereas a light but not significant reduction was induced in the late phase of the formalin test (Figure 3). After the DAMGO injection, we observed a reduction of the nociceptive behavior both early and late in the formalin test (Figure 3). The formalin early phase, which depends upon the direct excitement of sensory neurons through TRPA1 cation channel activation [43] of MORs at the peripheral endings of nociceptors, is responsible for meaningful analgesia [44], and it is not surprising that **K6** and DAMGO reduced formalin-induced nociception in the early phase of the test. The differences observed in the late phase are probably due to a different metabolic fate of **K6** and DAMGO after subcutaneous administration, depending upon the protease activity that might act in different ways on **K6** and DAMGO chemical structures.

### 3.4. Plasma Stability Results

**Figure 4:**
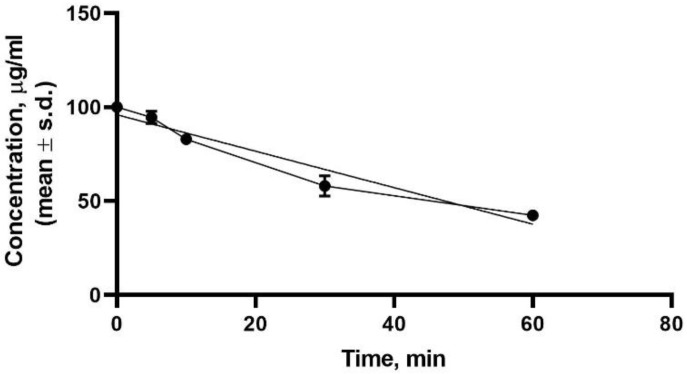
Stability of compound **K6** in human plasma. Concentration of K6 in μg/mL measured from 0 to 60 min in triplicate.

The plasma stability of compound **K6** was tested by incubation in human plasma at 37 °C. The degradation curve (Figure 4) was built by plotting the total amount of remaining peptide (expressed as μg/mL) vs. time (minutes). Concentration data were obtained in triplicate and analyzed as simple linear regression using GraphPad 8.3.1. The novel compound exhibits good stability in human plasma, showing a t_1/2_ = 47 min, according to the results obtained from the tail flick test after i.c.v. administration.

## 4. Conclusions

Chemical modification of endogenous opioid peptides promotes the development of novel analogs with increased potency and improved pharmacokinetic properties, e.g., the synthetic bivalent peptide biphalin enhanced stroke immunohistochemical and behavioral neuroprotection in comparison to DPDPE and DAMGO, reducing glutamate toxicity and oxidative stress [45,46,47,48]. Metabolites of the KP, especially kyna and L-kyn, play crucial roles in maintaining the normal brain function, preventing the over-activation of excitatory amino acid receptors, thus offering novel therapeutical opportunities for brain neuroprotection. In this work, we have synthesized six novel opioid analogs incorporating the L-kyn and kyna residues at different positions of several opioid peptide scaffolds. They were characterized by in vitro and in vivo assays to evaluate their ability to bind the NMDA/opioid receptors and to induce analgesic effects after i.c.v. and s.c. administration. These novel peptides do not bind to the NMDA receptors, and some of them showed good/high affinity for opioid receptors with different selectivity profiles. In particular, **KA1** exhibits the binding constant (Ki = 1.08) very close to that of DAMGO for the µ-opioid receptor and a pronounced selectivity but medium-low efficacy (E_max_ = 139%); thus the esterification of the ethanolamine portion with kyna does not add any particular advantage to the parent peptide DAMGO. On the other hand, the presence of L-kyn residue in place of native Phe in position 3 (**K2**) leads a potent and selective opioid fragment toward MOR (selectivity ration μ/δ/κ = 1:10,000:750), whereas the substitution in position 4 (**K3**) leads to a weak and unselective agonist at MOR and DOR. In the analogs **K4–K6**, the kyna residue was inserted in the fifth position of the YaGP peptide, with different *C*-terminus, respectively, methyl ester, free carboxylic acid, and amide. **K4** and **K5** show a similar mixed binding affinity for MOR and DOR, with a preference for MOR and none or weak affinity for KOR.

On the contrary, peptide **K6** shows an interesting behavior since it is able to bind all three opioid receptors with binding affinity ranging from high to modest ( Ki^μ^ = 1.84 nM, Ki^δ^ = 32.5 nM, Ki^κ^ = 127.7 nM), with a potency (logEC_50_ = −5.598) and efficacy at MOR (E_max_ = 211%) higher than that of DAMGO (E_max_ = 172%). Its activity on the GTPγS binding assay on rat brain membrane and guinea pig ileum is well antagonized by the co-administration of the selective antagonists for MOR and DOR at 10 μM concentration, prompting us to deeply investigate its anti-nociceptive effect in vivo. The formalin test, which is a model of inflammatory pain, revealed that the antinociceptive effect exerted by **K6** after subcutaneous administration is significant only in the early phase, whereas DAMGO is also active in the late phase. In the tail flick test, the **K6** analgesic profile after i.c.v. administration is superimposable to that of DAMGO, according to a good stability profile in human plasma. These data are encouraging to further develop opioid peptides containing kynurenine moieties since the insertion of kyna and kyn in our opioid model improved, in some cases, the binding affinity and was able to modulate the selectivity. Also, the possible role played by the metabolism of these peptides and their possible implication in different neuropathic and chronic pain models is unknown and worth further investigation.

## Figures and Tables

**Table 1:** Displacement of [^3^H]DAMGO, [^3^H]Ile^5,6^Delt II, [^3^H]HS665, and [^3^H]MK-801 by DAMGO, Ile^5,6^Delt II, HS665, MK-801, and oligopeptides in membranes of rat and guinea pig brains. The IC_50_ values for the MOR, DOR, KOR, and NMDA, according to the competition binding curves (see Figure S1), were converted into equilibrium inhibitory constant (K_i_) values using the Cheng–Prusoff [39] equation.

Ligand	K_i_ + S.E.M. (nM) Opioid System			NMDA System
	DAMGO ^a^	Ile^5,6^Delt II ^a^	HS665 ^b^	MK-801 ^a^
DAMGO	0.90 ± 0.28	n.d. ^c^	n.d. ^c^	n.d. ^c^
Ile^5,6^Delt II	n.d. ^c^	8.85 ± 0.77	n.d. ^c^	n.d. ^c^
HS665	n.d. ^c^	n.d. ^c^	2.38 ± 0.25	n.d. ^c^
MK-801	n.d. ^c^	n.d. ^c^	n.d. ^c^	11.45 ± 1.04
EM-2	3.16 ± 0.3	n.d. ^c^	n.d.^c^	n.d. ^c^
KYNA	n.d. ^c^	n.d. ^c^	n.d. ^c^	>10000
L-kyn	n.d. ^c^	n.d. ^c^	n.d. ^c^	>10000
KA1	1.08 ± 0.26	554.7 ± 0.8	>10000	>10000
K2	1.39 ± 0.30	>10000	1043 ± 0.3	>10000
K3	197.3 ± 0.36	158.8 ± 1.6	>10000	>10000
K4	2.29 ± 0.28	31.2 ± 0.7	>10000	>10000
K5	9.11 ± 0.32	94.4 ± 0.8	>10000	>10000
K6	1.84 ± 0.27	32.5 ± 0.8	127.7 ± 0.3	>10000

**^a^** rat brain membrane, **^b^** guinea pig brain membrane, **^c^** not determined.

**Table 2:** G-protein activation DAMGO, and novel oligopeptides in [^35^S]GTPγS binding assays using rat brain membrane homogenates. The values were calculated according to dose-response binding curves.

Ligand	Maximal Stimulation (Efficacy)	Potency
	E_max_ ± S.E.M. (%)	Log EC_50_ ± S.E.M.
DAMGO	172.0 ± 3.5	−6.384 ± 0.101
KA1	140.9 ± 1.4	−6.504 ± 0.076
K2	121.6 ± 2.5	−7.535 ± 0.354
K3	114.0 ± 2.1	−6.993 ± 0.422
K4	155.5 ± 4.8	−6.073 ± 0.172
K5	149.2 ± 3.5	−5.990 ± 0.111
K6	209.7 ± 3.4	−5.984 ± 0.054

**Table 3:** The maximal G-protein efficacy (E_max_) of novel oligopeptides in the absence or presence of the selective MOR antagonist cyprodime and the selective DOR antagonist naltrindole in rat brain membrane homogenates and in the absence or presence of the selective KOR antagonist norbinaltorphine in guinea pig brain membrane homogenates in [^35^S]GTPγS binding assays. The values were calculated according to bar graphs in Figure S3.

	Ligand	MOR	DOR	Ligand	KOR
		Ligand + Cyp	Ligand + NTI		Ligand + Nor-BNI
	E_max_ + S.E.M. (%)				
KA1	139.6 ± 8.2	94.7 ± 3.7 **	102.5 ± 1.3 *	111.7 ± 1.1	100.7 ± 6.2 ^ns^
K2	129.9 ± 8.2	88.0 ± 3.3 **	88.8 ± 1.9 **	92.8 ± 2.0	100.7 ± 6.0 ^ns^
K3	118.9 ± 3.0	98.5 ± 1.2 **	97.8 ± 1.7 **	95.2 ± 2.2	106.0 ± 6.3 ^ns^
K4	160.6 ± 5.7	101.6 ± 2.6 ***	103.6 ± 3.2 ***	125.2 ± 2.0	104.9 ± 9.5 *
K5	153.1 ± 5.9	108.8 ± 1.6 **	103.8 ± 1.9 ***	106.9 ± 0.3	110.5 ± 5.0 ^ns^
K6	211.7 ± 3.1	108.5 ± 2.4 ***	113.3 ± 2.9 ***	139.8 ± 2.3	142.1 ± 7.1 ^ns^

Experimental data were processed by GraphPad Prism 5.0 using bar graphs. ns: not significant; * *p* < 0.05; ** *p* < 0.01; *** *p* < 0.001 based on unpaired *t*-tests.

## Supplementary Materials

The following are available online at https://www.mdpi.com/2218-273X/10/2/284/s1, Figure S1: MOR (A), DOR (B), KOR (C) and NMDA (D) binding affinity of the novel oligopeptides, Figure S2: The effect of oligopeptides on G-protein activity compared to DAMGO in [^35^S]GTPγS binding assay in rat brain membrane homogenates, Figure S3: The effect of novel oligopeptides on G-protein activity in [^35^S]GTPγS binding assays in the absence or presence of the selective MOR antagonist cyprodime (Cyp) and the selective DOR antagonist naltrindole (NTI) in rat brain membrane homogenates (Figure A) and the selective KOR antagonist norbinaltorphine (nor-BNI) in guinea pig brain membrane homogenates (Figure B).

## Abbreviations

KP	kynurenine pathway
kyn	kynurenine
kyna	kynurenic acid
FDA	Food and Drug Administration
CNS	central nervous system
BBB	blood brain barrier
GPR35	G protein-coupled receptor 35
SVCT2	Sodium-dependent vitamin C transporter 2
MOR	μ-opioid receptor; DOR, δ-opioid receptor
DOR	δ-opioid receptor
KOR	k-opioid receptor; GPCRs, G protein coupled receptors
GPCRs	G protein coupled receptors
NMR	Nuclear magnetic resonance
TFA	trifloroacetic acid
ACN	acetonitrile
RP-HPLC	Reverse Phase High performance liquid chromatography
TMS	trimethylsilane
ESI	Electrospray ionization
LRMS	Low Resolution Mass Spectroscopy
HOBt	1-hydroxybenzotriazole
DMAP	4-Dimethylaminopyridine
EDC^.^HCl	1-Ethyl-3-(3-dimethylaminopropyl)carbodiimide hydrochloride
EtOAc	ethyl acetate
THF	tetrahydrofurane
NMM	N-methylmorpholine
iBCF	isobuthylchloroformiate
DMSO	dimethylsulfoxide
Boc	tert-butyloxycarbonyl
Ar	aryl
EM-2	endomorphine-2
BSA	Bovine serum albumin
i.c.v.	intracerebroventricular
Cyp	cyprodime
NTI	naltrindole
Nor-BNI	norbinaltorphine
DPDPE	[D-Pen,D-Pen5]Enkephalin
DIPEA	N,N-Diisopropylethylamine
DMF	dimethylformamide
DCM	dichloromethane
MeOH	methanol
TIPS	triisopropylsilane
EGTA	ethylene glycol-bis(2-aminoethylether)-N,N,N′,N′-tetraacetic acid
GDP	guanosine 5’-diphosphate
GTP	guanosine-5’-triphosphate
DMSO	dimethylsulfoxide
DAMGO	[D-Ala^2^, N-MePhe^4^, Gly-ol]-enkephalin
IleDelt II	Ile^5,6^-deltorphin II
[^35^S]GTPγS	guanosine-5’-[^35^S]thiophosphate
NMDA	N-methyl-D-aspartate receptor
Tris-HCl	tris-(hydroxymethyl)-aminomethane hydrochloride
