# Supplementary material for: Discovery of Kynurenines Containing Oligopeptides as Potent Opioid Receptor Agonists
Source: Biomolecules. 2020 Feb 12;10(2):284. doi: 10.3390/biom10020284 (PMC7072329; doi:10.3390/biom10020284)
Supplement: Supplementary file 1 [file biomolecules-10-00284-s001.pdf]

SUPPORTING MATERIALS

Discovery of Kynurenines containing oligopeptides as potent opioid receptor agonists

Edina Szűcs,[a,b] Azzurra Stefanucci,[c,*] Marilisa Pia Dimmito,[c] Ferenc Zádor,[a] Stefano Pieretti,[d] Gokhan Zengin,[e] László Vécsei,[f] Sándor Benyhe,[a] Marianna Nalli,[g] Adriano Mollica[c]

[a] Institute of Biochemistry, Biological Research Center, Hungarian Academy of Sciences, H-6726 Szeged, Temesvári krt. 62., Hungary.

[b] Doctoral School of Theoretical Medicine, Faculty of Medicine, University of Szeged, Dóm tér 10, H-6720 Szeged, Hungary.

[c] Department of Pharmacy, University of Chieti-Pescara "G. d'Annunzio", Via dei Vestini 31, 66100 Chieti, Italy.

[d] National Center for Drug Research and Evaluation, Istituto Superiore di Sanità, Viale Regina Elena 299, 00161, Rome. Italy.

[e] Department of Biology, Science Faculty, Selcuk University, 42250 Konya, Turkey.

[f] Department of Neurology, Interdisciplinary Excellence Centre, Faculty of Medicine, University of Szeged, MTA-SZTE Neuroscience Research Group, H-6725 Szeged, Hungary.

[g] Laboratory affiliated with the Institute Pasteur Italy-Cenci Bolognetti Foundation, Department of Drug Chemistry and Technologies, Sapienza University of Rome, Piazzale Aldo Moro 5, I-00185, Roma, Italy.

*Correspondence: email: a.stefanucci@unich.it

Table of contents

	Pages
Compounds characterization	2-8
RP-HPLC analytical traces of peptides **KA1, K2-K6**	9,10
LRMS spectra of **KA1, K2-K6**	11-16
^{1}H NMR spectra of final compounds	17-22
Binding affinity curves of peptides **KA1, K2-K6**	23
GTP binding assay data of peptides **KA1, K2-K6**	24,25
Plasma stability assay	26
References	27

Compounds characterization

Boc-*N*-aminoethanol (**1**): The synthesis of Boc-*N*-aminoethanol has been performed following the procedure described by Ghilardi *et al.*[1]. The desired product has been obtained in quantitative yield, characterization data are in agreement with those reported in literature.

Boc-*N*-aminoethanol-kynurenic acid ester (**2**): Boc-*N*-aminoethanol (322.2 mg, 2 mmol) has been reacted with the kynurenic acid (378.3 mg, 2 mmol) following the general procedure. The crude product has been purified by silica gel column chromatography (from EtOAc : n-hexane = 80:20 to 90:10) to obtain compound **2** in 60% yield. R*f* = 0.55 (EtOAc 100%); ^{1}H-NMR (DMSO-d_6) δ: 12.00 (s, 1H, OH), 8.07 (d, 1H, kyna ArH), 7.91 (d, 1H, kyna ArH), 7.69 (t, 1H, kyna ArH), 7.35 (t, 1H, kyna ArH), 7.16 (t, 1H, N*H* Boc), 6.75 (s, 1H, kyna ArH), 4.31 (t, 2H, CH_2-O), 3.48 (q, 2H, $C\underline{H}_2$-NH), 1.34 (s, 9H, 3*CH_3).

Boc-*N*(Me)Phe-aminoethanol-kynurenic acid (**3**): Intermediate **2** has been deprotected with a mixture of TFA:DCM =1:1 at r.t. for 1h, then the solvent was evaporated in rotavapor and the residue washed with DCM (3 times), dried in rotary evaporator and high vacuum. The so obtained TFA salt was coupled with Boc-*N*(Me)Phe-OH (132.2 mg, 0.5 mmol) following the general procedure of coupling. The crude white product has been used for the next coupling reaction without further purification (158 mg, 75% yield); R*f* = 0.53 (EtOAc : MeOH = 98:2). ^{1}H-NMR (DMSO-d_6) δ: 12.00 (s, 1H, OH), 8.21 (t, 1H, N*H*-CH_2), 8.06 (d, 1H, kyna ArH), 7.91 (d, 1H, kyna ArH), 7.69 (t, 1H, kyna ArH), 7.35 (t, 1H, kyna ArH), 7.23-7.17 (m, 5H, Phe ArH), 6.74 (s, 1H, kyna ArH), 4.38-4.33 (m, 3H, CH_2-O, Phe H^α), 3.48 (q, 2H, $C\underline{H}_2$-NH), 3.19-3.10 (m, 1H, Phe H^β), 2.84-2.77 (m, 1H, Phe H^β), 2.60 (s, 3H, NCH_3), 1.11 (s, 9H, 3*CH_3).

Boc-Gly-*N*(Me)Phe-aminoethanol-kynurenic acid (**4**): Intermediate **3** has been deprotected with a mixture of TFA:DCM =1:1 at r.t. for 1h, then the solvent was evaporated in rotavapor and the residue washed with DCM (3 times), dried in rotary evaporator and high vacuum. The so obtained TFA salt was coupled with Boc-Gly-OH (61.7 mg, 0.3 mmol) following the general coupling procedure. The crude product has been used for the next coupling reaction without further purification (123 mg, 70% yield); R*f* = 0.28 (EtOAc : MeOH = 98:2). ^{1}H-NMR (DMSO-d_6) δ: 12.03 (s, 1H, OH), 8.22 (t, 1H, N*H*-CH_2), 8.07 (d, 1H, CH Ar), 7.92 (d, 1H, kyna ArH), 7.69 (t, 1H, kyna ArH), 7.35 (t, 1H, kyna ArH), 7.21-7.08 (m, 6H, Phe ArH, N*H*-Boc), 6.73 (s, 1H, kyna ArH), 5.12 (q, 1H, Phe H^α), 4.39 (d, 2H, Gly H^α), 4.03-3.93 (m, 2H, CH_2-O), 3.50-3.38 (m, 2H, Phe H^β), 2.81 (s, 3H, NCH_3), 1.32 (s, 9H, 3*CH_3).

Boc-DAla-Gly-*N*(Me)Phe-aminoethanol-kynurenic acid (**5**): Intermediate **4** has been deprotected with a mixture of TFA:DCM =1:1 at r.t. for 1h, then the solvent was evaporated in rotavapor and the residue washed with DCM (3 times), dried in rotary evaporator and high vacuum. The so obtained TFA salt was coupled with Boc-DAla-OH (46.5 mg, 0.2 mmol) following the general procedure of coupling. The crude product has been purified by silica gel column chromatography (from EtOAc: n-

hexane = 90:10 to 98:2) to give product **5** in 80% yield (111 mg); R*f* = 0.22 (EtOAc : MeOH = 98:2). ^{1}H-NMR (DMSO-d$_6$) δ: 12.01 (s, 1H, OH), 8.22 (t, 1H, N*H*-CH$_2$), 8.07 (d, 1H, kyna ArH), 7.92 (d, 1H, kyna ArH), 7.69 (t, 1H, kyna ArH), 7.35 (t, 1H, kyna ArH), 7.21-7.01 (m, 7H, Phe ArH, NH-Boc, Gly NH), 6.73 (s, 1H, kyna ArH), 5.12 (q, 1H, Phe H$^\alpha$), 4.34 (d, 2H, Gly H$^\alpha$), 4.03-3.93 (m, 3H, CH$_2$-O, DAla H$^\alpha$), 3.50-3.38 (m, 2H, Phe H$^\beta$), 2.81 (s, 3H, NCH$_3$), 1.32 (s, 9H, 3*CH$_3$), 1.09 (d, 3H, CH$_3$ DAla).

Boc-Tyr-DAla-Gly-*N*(Me)Phe-aminoethanol-kynurenic acid (**6**): Intermediate **5** has been deprotected with a mixture of TFA:DCM =1:1 at r.t. for 1h, then the solvent was evaporated in rotavapor and the residue washed with DCM, dried in rotary evaporator repetitively and high vacuum. The so obtained TFA salt was coupled with Boc-Tyr-OH (53.3 mg, 0.2 mmol) following the general procedure of coupling. The crude product has been purified by silica gel column chromatography (from EtOAc 100% to EtOAc : MeOH = 95:5) to give product **6** in 66% yield (104 mg); R*f* = 0.18 (EtOAc : MeOH = 98:2). ^{1}H-NMR (DMSO-d$_6$) δ: 12.01 (s, 1H, OH), 9.13 (s, 1H, OH Tyr), 8.07 (d, 1H, kyna ArH), 7.95 (d, 1H, kyna ArH), 7.69 (t, 1H, kyna ArH), 7.35 (t, 1H, kyna ArH), 8.20 (t, 1H, N*H*-CH$_2$), 8.07 (d, 1H, CH Ar), 7.97-7.90 (m, 2H, CH Ar, NH DAla), 7.69 (t, 1H, CH Ar), 7.35 (t, 1H, CH Ar), 7.21-7.09 (m, 6H, Phe ArH, NH Gly), 6.98 (d, 2H, Tyr ArH), 6.82 (d, 1H, N*H*-Boc), 6.74 (s, 1H, CH Ar), 6.74 (s, 1H, kyna ArH), 6.59 (d, 2H, Tyr ArH), 5.09 (q, 1H, Phe H$^\alpha$), 4.31-4.25 (m, 3H, CH$_2$-O, Tyr H$^\alpha$), 4.03-3.94 (m, 3H, Gly H$^\alpha$, DAla H$^\alpha$), 3.48-3.15 (m, 2H, Phe H$^\beta$), 2.89-2.58 (m, 2H, Tyr H$^\beta$), 2.75 (s, 3H, NC*H*$_3$), 1.26 (s, 9H, 3*CH$_3$), 1.14 (d, 3H, CH$_3$ DAla).

TFA·NH$_2$-Tyr-DAla-Gly-*N*(Me)Phe-aminoethanol-kynurenic acid (**KA1**): Boc-protected peptide **6** has been deprotected with a mixture of TFA:DCM =1:1 at r.t. for 1h, then the solvent was evaporated in rotavapor and the oily residue washed with DCM (3 times), dried in rotary evaporator and high vacuum to give product **KA1** as TFA salt, in 71% overall yield after RP-HPLC purification; (R_t = 16.02 min). ^{1}H-NMR (DMSO-d$_6$) δ: 12.05 (s, 1H, OH), 9.31 (s, 1H, OH Tyr), 8.48 (d, 1H, *J* = 7.8 Hz, DAla NH), 8.20 (t, 1H, N*H*-CH2), 8.14 (t, 1H, Gly NH), 8.05 (m, 4H, NH_3^+, kyna ArH), 7.93 (d, 1H, *J* = 8.7 Hz, kyna ArH), 7.70 (d, 1H, *J* = 7.5 Hz, kyna ArH), 7.36 (t, 1H, *J* = 7.5 Hz, kyna ArH), 7.22-7.10 (m, 5H, Phe ArH), 6.99 (d, 2H, *J* = 8.4 Hz, Tyr ArH), 6.74 (s, 1H, kyna ArH), 6.67 (d, 2H, *J* = 8.4 Hz, Tyr ArH), 5.09 (q, 1H, *J* = 9.9 Hz, Phe H$^\alpha$), 4.45-4.31 (m, 3H, CH$_2$-O, Tyr H$^\alpha$), 4.01-3.90 (m, 2H, Gly H$^\alpha$), 3.44-3.17 (m, 2H, Phe H$^\beta$), 2.95-2.75 (m, 2H, Tyr H$^\beta$), 2.82 (s, 3H, NCH$_3$), 1.01 (d, 3H, *J* = 6.6 Hz, CH$_3$ DAla). LRMS for $C_{36}H_{40}N_6O_8$ calcd. m/z = 684.2, found 685.8 $[M+H]^+$.

Boc-Kynurenine (**7**): This intermediate has been prepared following the procedure reported by Tsentalovich *et al.*[2] and it has been previously characterized in literature.

Boc-Kynurenine-Phe-NH$_2$ (**8**): H-Phe-NH$_2$ has been prepared starting from natural amino acid Boc-Phe-OH, following the general procedure of amidation. The so obtained product (91 mg, 0.3 mmol) has been deprotected with a mixture of TFA:DCM =1:1 at r.t. for 1h, then the solvent was evaporated in rotavapor and the residue washed with DCM (3 times), dried in rotary evaporator and high vacuum.

Coupling reaction with Boc-Kyn-OH (116.7, 0.4 mmol) has been performed following the general procedure, to give intermediate peptide **8** in 61% yield (95 mg); R*f* = 0.5 (EtOAc 100%). ^{1}H NMR (DMSO-d$_6$) δ: 8.63 (d, 1H, kyn NH), 7.77 (dd, 1H, kyn ArH), 7.61 (d, 1H, Phe NH), 7.26-7.14 (m, 19 H, Phe ArH, kyn ArH, Boc-N*H*, Phe NH$_2$), 6.60-6.53 (m, 4H, kyn NH$_2$, kyn ArH), 4.58 (q, 1H, Phe H$^\alpha$), 4.35 (m, 1H, kyn H$^\alpha$), 3.05-2.65 (m, 4H, Phe H$^\beta$, kyn H$^\beta$), 1.26 (s, 9H, 3*CH$_3$).

Boc-Pro-Kyn-Phe-NH$_2$ (**9**): Intermediate compound **8** has been deprotected with a mixture of TFA:DCM =1:1 at r.t. for 1h, then the solvent was evaporated in rotavapor and the residue washed with DCM (3 times), dried in rotary evaporator and high vacuum and allowed to react with Boc-Pro-OH (49.5, 0.2 mmol) following the general procedure of coupling. Intermediate peptide **9** has been obtained in 81% yield (93 mg); R*f* = 0.43 (EtOAc 100%). ^{1}H NMR (DMSO-d$_6$) δ: 8.68 (d, 1H, kyn NH), 7.79 (d, 1H, kyn ArH), 7.61 (d, 1H, Phe NH), 7.26-7.14 (m, 9H, Phe ArH, kyn ArH, Boc-N*H*, Phe NH$_2$), 6.60-6.53 (m, 4H, kyn NH$_2$, kyn ArH), 4.58 (q, 1H, Phe H$^\alpha$), 4.35-4.27 (m, 2H, Pro H$^\alpha$, kyn H$^\alpha$), 3.05-2.76 (m, 6H, Phe H$^\beta$, kyn H$^\beta$, Pro H-1), 1.88-1.83 (m, 4H, Pro H-2, H-3), 1.25 (s, 9H, 3*CH$_3$).

Boc-Tyr-Pro-Kyn-Phe-NH$_2$ (**10**): Intermediate compound **9** has been deprotected with a mixture of TFA:DCM =1:1 at r.t. for 1h, then the solvent was evaporated in rotavapor and the residue washed with DCM (3 times), dried in high vacuum and allowed to react with Boc-Tyr-OH (52.2 mg, 0.2 mmol) following the general procedure of coupling. The Boc-protected peptide **10** has been obtained in 73% yield (88.7 mg); R*f* = 0.15 (EtOAc 100%). ^{1}H NMR (DMSO-d$_6$) δ: 9.20 (s, 1H, Tyr OH), 8.63 (d, 1H, kyn NH), 7.77 (dd, 1H, kyn ArH), 7.61 (d, 1H, Phe NH), 7.26-7.14 (m, 11 H, Phe ArH, Tyr ArH, kyn ArH, Boc-N*H*, Phe NH$_2$), 6.73 (d, 2H, Tyr ArH), 6.60-6.53 (m, 4H, kyn NH$_2$, Kyn ArH), 4.58 (q, 1H, Phe H$^\alpha$), 4.35-4.22 (m, 3H, Pro H$^\alpha$, kyn H$^\alpha$, Tyr H$^\alpha$), 3.59-3.39 (m, 2H, Tyr H$^\beta$), 3.05-2.54 (m, 6H, Phe H$^\beta$, kyn H$^\beta$, Pro H-1), 1.88-1.83 (m, 4H, Pro H-2, H-3), 1.25 (s, 9H, 3*CH$_3$).

TFA·NH$_2$-Tyr-Pro-Kyn-Phe-NH$_2$ (**K2**): Intermediate peptide **10** has been deprotected with a mixture of TFA:DCM =1:1 at r.t. for 1h, then the solvent was evaporated in rotavapor and the residue washed with DCM (3 times), dried in high vacuum. The so obtained crude product has been purified on RP-HPLC to give final product as TFA salt in 72% overall yield; R$_t$ = 15.38 min. ^{1}H NMR (DMSO-d$_6$) δ: 9.35 (m, 5H, Tyr OH, NH$_3^+$, kyn NH), 8.35 (dd, 1H, kyn ArH), 8.23 (dd, 1H, kyn ArH), 7.98 (dd, 1H, kyn ArH), 7.88 (d, 1H, *J* = 8.1 Hz, Phe NH), 7.66 (s, 2H, Phe NH$_2$), 7.31-7.22 (m, 8H, Phe ArH, Tyr ArH, kyn ArH), 6.90 (d, 2H, *J* = 8.4 Hz, Tyr ArH), 6.69-6.55 (m, 2H, kyn NH$_2$), 4.68 (q, 1H, Phe H$^\alpha$), 4.37-4.20 (m, 3H, Pro H$^\alpha$, kyn H$^\alpha$, Tyr H$^\alpha$), 3.59-3.39 (m, 2H, Tyr H$^\beta$), 3.02-2.70 (m, 6H, Phe H$^\beta$, kyn H$^\beta$, Pro H-1), 1.88-1.83 (m, 4H, Pro H-2, H-3). LRMS for $C_{33}H_{38}N_6O_6$ calcd. m/z = 614.2, found 615.0 [M+H]$^+$.

Boc-Kyn-NH$_2$ (**11**): Intermediate **7** has been converted in its amide derivative **11**, following the general procedure of amidation (71% yield, 106 mg); R*f* = 0.6 (EtOAc 100%). ^{1}H NMR (DMSO-d$_6$) δ: 7.67 (d, 1H, kyn ArH), 7.24-7.17 (m, 4H, kyn ArH, 1 kyn NH$_2$, kyn CO-NH$_2$), 7.01 (brs, 1H, kyn CO-NH$_2$), 6.86 (d, 1H, NHBoc), 6.72 (d,

1H, kyn ArH), 6.48 (t, 1H, kyn ArH), 4.38 (q, 1H, kyn H^{α}), 3.21-3.13 (m, 2H, kyn H^{β}), 1.34 (s, 9H, 3*CH_3).

Boc-Phe-Kyn-NH_2 (**12**): The intermediate compound **11** has been treated with a mixture of TFA:DCM =1:1 at r.t. for 1h, then the solvent was evaporated in rotavapor and the residue washed with DCM (3 times), dried in rotary evaporator and coupled with Boc-Phe-OH (96.8 mg, 0.4 mmol), following the general procedure. Compound **12** has been obtained quantitatively (150.8 mg). R*f* = 0.6 (EtOAc 100%). ^{1}H NMR (DMSO-d_6) δ: 7.91 (t, 1H, kyn ArH), 7.69 (d, 1H, kyn ArH), 7.24-7.16 (m, 9H, Phe ArH, kyn NH_2*2), 7.69 (d, 1H, Phe NH), 6.76 (t, 1H, Boc-N*H*), 6.61 (d, 2H, 2* kyn ArH), 4.61 (q, 1H, Phe H^{α}), 4.41 (m, 1H, kyn H^{α}), 3.36-2.98 (m, 4H, Phe H^{β}, kyn H^{β}), 1.26 (s, 9H, 3*CH_3).

Boc-Pro-Phe-Kyn-NH_2 (**13**): The intermediate compound **12** has been treated with a mixture of TFA:DCM =1:1 at r.t. for 1h, then the solvent was evaporated in rotavapor and the residue washed with DCM (3 times), dried in rotary evaporator and coupled with Boc-Pro-OH (77.7 mg, 0.4 mmol), following the general procedure. Compound **13** has been obtained in 72% yield after trituration in Et_2O (130 mg); R*f* = 0.26 (EtOAc 100%). ^{1}H NMR (DMSO-d_6) δ: 8.08 (d, 1H, NH Phe), 7.95 (t, 1H, kyn ArH), 7.69 (d, 1H, kyn ArH), 7.25-7.10 (m, 9H, Phe ArH, kyn NH_2*2), 6.73 (d, 2H, Tyr ArH), 6.52 (d, 2H, 2*kyn ArH), 4.61 (q, 1H, Phe H^{α}), 4.49 (m, 1H, kyn H^{α}), 4.39 (m, 1H, Pro H^{α}) 3.02-2.97 (m, 6H, Phe H^{β}, kyn H^{β}, Pro H-1), 2.05-1.85 (m, 4H, Pro H-2, H-3), 1.21 (s, 9H, 3*CH_3).

Boc-Tyr-Pro-Phe-Kyn-NH_2 (**14**): The intermediate compound **13** has been treated with a mixture of TFA:DCM =1:1 at r.t. for 1h, then the solvent was evaporated in rotavapor and the residue washed with DCM (3 times), dried in rotary evaporator and coupled with Boc-Tyr-OH (72.9 mg, 0.2 mmol), following the general procedure. Boc-protected peptide **14** has been obtained in 95% yield after trituration in Et_2O (159 mg); R*f* = 0.55 (EtOAc:MeOH = 98:2). ^{1}H NMR (DMSO-d_6) δ: 9.15 (s, 1H, Tyr OH), 7.97 (t, 1H, kyn ArH), 7.69 (d, 1H, kyn ArH), 7.24-7.13 (m, 9H, Phe ArH, kyn NH_2*2), 7.03-6.90 (m, 3H, Tyr ArH, Phe NH), 6.72 (d, 2H, Tyr ArH), 6.61 (d, 2H, 2* kyn ArH), 6.46 (t, 1H, Boc-N*H*), 4.61 (q, 1H, Phe H^{α}), 4.40-4.20 (m, 2H, kyn H^{α}, Tyr H^{α}), 3.56-3.46 (m, 2H, Tyr H^{β}), 3.03-2.55 (m, 6H, Phe H^{β}, kyn H^{β}, Pro H-1), 1.96-1.70 (m, 4H, Pro H-2, H-3), 1.26 (s, 9H, 3*CH_3).

TFA·NH_2-Tyr-Pro-Phe-Kyn-NH_2 (**K3**): Intermediate peptide **14** has been deprotected with a mixture of TFA:DCM =1:1 at r.t. for 1h, then the solvent was evaporated in rotavapor and the residue washed with DCM (3 times), dried in rotary evaporator and high vacuum. The so obtained crude product has been purified on RP-HPLC to give final product **K3** as TFA salt in 83% overall yield; R_t = 15.50 min. ^{1}H NMR (DMSO-d_6) δ: 9.43 (m, 4H, Tyr OH, NH_3^+ Tyr), 7.97 (t, 1H, kyn ArH), 7.69 (d, 1H, kyn ArH), 7.24-7.13 (m, 9H, Phe ArH, kyn NH_2*2), 7.03-6.90 (m, 3H, Tyr ArH, Phe NH), 6.72 (d, 2H, J = 8.1 Hz, Tyr ArH), 6.61 (d, 2H, J = 8.7 Hz, 2* kyn ArH), 6.46 (t, 1H, Boc-N*H*), 4.61 (q, 1H, Phe H^{α}), 4.40-4.20 (m, 2H, kyn H^{α}, Tyr H^{α}), 3.56-3.46 (m,

2H, Tyr H^{β}), 3.03-2.55 (m, 6H, Phe H^{β}, kyn H^{β}, Pro H-1), 1.96-1.70 (m, 4H, Pro H-2, H-3), 1.26 (s, 9H, 3*CH_3). LRMS for $C_{33}H_{38}N_6O_6$ calcd. m/z = 614.2, found 615.7 $[M+H]^+$.

L-Kyn-OCH_3 (**15**): L-Kynurenine (50 mg, 0.2 mmol) was converted in its methyl ester derivative **15**, following the procedure described in Stefanucci *et al.*[3]. The desired product has been obtained quantitatively (53 mg). ^1H NMR (DMSO-d_6) δ: 8.55 (brs, 4H, 2*NH_2), 7.70 (d, 1H, kyn ArH), 7.30 (t, 1H, kyn ArH), 6.77 (d, 1H, kyn ArH), 6.56 (t, 1H, kyn ArH), 4.38 (m, 1H, kyn H^{α}), 3.69 (m, 5H, kyn H^{β}, -OCH_3).

Boc-Phe-Kyn-OCH_3 (**16**): Intermediate **15** was used for the next coupling reaction with BocPhe-OH (58.4 mg, 0.2 mmol), following procedure. The crude product has been purified by silica gel column chromatography (EtOAc : n-hexane = 80:20), to give intermediate **16** in 60% yield (75 mg); R*f* = 0.85 (EtOAc 100%). ^1H NMR (DMSO-d_6) δ: 8.24 (d, 1H, kyn NH-CO), 7.70 (d, 1H, kyn ArH), 7.28-7.13 (m, 8H, kyn ArH, kyn NH_2), 6.92 (d, 1H, N*H*-Boc), 6.74 (d, 1H, kyn ArH), 6.52 (t, 1H, kyn ArH), 4.76 (q, 1H, Phe H^{α}), 4.16 (q, 1H, kyn H^{α}), 3.58 (s, 3H, -OCH_3), 3.44-3.39 (m, 1H, kyn H^{β}), 2.97-2.63 (m, 3H, 1H kyn H^{β}, 2H Phe H^{β}), 1.24 (s, 9H, 3*CH_3).

Boc-Gly-Phe-Kyn-OCH_3 (**17**): Intermediate **16** has been treated with a mixture of TFA:DCM = 1:1 at r.t. for 1h, then DCM was removed in rotavapor and the crude residue dried in high vacuum. The so obtained compound was used for the next coupling reaction with BocGly-OH (17.5 mg, 0.1 mmol) without further purification, following the general procedure. The desired peptide **17** has been obtained in quantitative yield after reaction work-up (46.9 mg); R*f* = 0.71 (EtOAc 100%). ^1H NMR (DMSO-d_6) δ: 8.50 (1H, d, kyn NH-CO), 7.88 (1H, d, Phe NH), 7.69 (d, 1H, kyn ArH), 7.26-7.13 (m, 9H, NH Phe, kyn ArH, kyn NH_2), 6.89 (t, 1H, NH-Boc), 6.73 (d, 1H, kyn ArH), 6.52 (t, 1H, kyn ArH), 4.75 (q, 1H, Phe H^{α}), 4.53 (m, 1H, kyn H^{α}), 3.72-3.44 (m, 6H, Gly H^{α}, -OCH_3, 1 Phe H^{β}), 2.98-2.69 (m, 3H, kyn H^{β}, 1 Phe H^{β}).

Boc-DAla-Gly-Phe-Kyn-OCH_3 (**18**): Intermediate **17** has been treated with a mixture of TFA:DCM = 1:1 at r.t. for 1h, then DCM was removed in rotavapor and the crude residue dried in high vacuum. The so obtained compound was used for the next coupling reaction with Boc-DAla-OH (20.8 mg, 0.1 mmol) without further purification, following the general procedure. The crude product has been purified by silica gel column chromatography (from EtOAc : n-hexane = 80:20 to 90:10), to give intermediate **16** in 84% yield (50 mg); R*f* = 0.32 (EtOAc 100%). ^1H NMR (DMSO-d_6) δ: 8.45 (1H, d, kyn NH-CO), 8.03 (d, 1H, kyn ArH), 7.92 (t, 1H, Gly NH), 7.69 (d, 1H, kyn ArH), 7.26-7.13 (m, 8H, Phe ArH, Kyn NH_2, Phe NH), 6.95 (d, 1H, NH-Boc), 6.73 (d, 1H, kyn ArH), 6.52 (t, 1H, kyn ArH), 4.75 (q, 1H, Phe H^{α}), 4.53 (m, 1H, kyn H^{α}), 3.91 (m, 1H, DAla H^{α}), 3.78-3.44 (m, 5H, Gly H^{α}, -OCH_3), 3.39 (d, 1H, Phe H^{β}), 2.98-2.69 (m, 3H, kyn H^{β}, 1 Phe H^{β}), 1.33 (s, 9H, 3*CH_3), 1.09 (d, 3H, DAla H^{β}).

Boc-Tyr-DAla-Gly-Phe-Kyn-OCH_3 (**19**): Intermediate **18** has been treated with a mixture of TFA:DCM = 1:1 at r.t. for 1h, then DCM was removed in rotavapor and the crude residue dried in high vacuum. The so obtained compound was used for the next coupling reaction with Boc-Tyr-OH (31.2 mg, 0.1 mmol) without further purification, following the general procedure, to give Boc-protected peptide **19** in

72% yield after trituration in Et_2O (48 mg); R*f* = 0.22 (EtOAc 100%). ^{1}H NMR (DMSO-d_6) δ: 9.14 (s, 1H, OH Tyr), 8.45 (1H, d, kyn NH-CO), 8.03 (m, 2H, DAla NH, Gly NH), 7.69 (d, 1H, kyn ArH), 7.23-7.14 (m, 8H, Phe ArH, kyn NH_2, kyn ArH), 6.97 (d, 2H, Tyr ArH), 6.89 (d, 1H, NH-Boc), 6.74 (d, 1H, kyn ArH), 6.59 (d, 2H, Tyr ArH), 4.75 (q, 1H, Phe H^α), 4.54 (m, 1H, kyn H^α), 4.28 (quint, 1H, DAla H^α), 3.95 (m, 1H, Tyr H^α), 3.65-3.59 (m, 5H, Gly H^α, -OCH_3), 3.39 (m, 2H, Phe H^β), 3.05 (m, 2H, kyn H^β), 2.99-2.70 (m, 2H, Tyr H^β), 1.26 (s, 9H, 3*CH_3), 1.06 (d, 3H, DAla H^β).

TFA·NH_2-Tyr-DAla-Gly-Phe-Kyn-OCH_3 (**K4**): Intermediate peptide **19** has been deprotected with a mixture of TFA:DCM =1:1 at r.t. for 1h, then the solvent was evaporated in rotavapor and the residue washed with DCM (3 times), dried in high vacuum. The so obtained crude product has been purified on RP-HPLC to give final product **K4** as TFA salt in 62% overall yield; R_t = 16.30 min. ^{1}H NMR (DMSO-d_6) δ: 9.33 (s, 1H, OH Tyr), 8.60-8.54 (m, 2H, NH-CO, Phe NH), 8.19 (t, 1H, *J* = 5.4 Hz, Gly NH), 8.01 (brs, 3H, Tyr NH_3^+), 7.70 (d, 1H, *J* = 8.4 Hz, kyn ArH), 7.27-7.14 (m, 9H, Phe ArH, kyn NH_2, kyn ArH), 6.99 (d, 1H, *J* = 8.4 Hz, Tyr ArH), 6.74 (d, 1H, *J* = 9 Hz, DAla NH), 6.67 (d, 1H, *J* = 8.1 Hz, Tyr ArH), 6.52 (t, 1H, *J* = 7.8 Hz, kyn ArH), 4.75 (q, 1H, *J* = 7.5 Hz, Phe H^α), 4.54 (m, 1H, J = 7.2 Hz, kyn H^α), 4.28 (quint, 1H, DAla H^α), 3.95 (m, 1H, Tyr H^α), 3.65-3.59 (m, 7H, Gly H^α, -OCH_3, 1 Phe H^β, 1 Tyr H^β), 2.99-2.70 (m, 4H, 1 Phe H^β, 1 Tyr H^β, kyn H^β), 1.02 (d, 3H, *J* = 6.9 Hz, DAla H^β). LRMS for $C_{34}H_{40}N_6O_8$ calcd. m/z = 660.2, found 685.8 $[M+Na]^+$.

Boc-Tyr-DAla-Gly-Phe-Kyn-OH (**20**): Intermediate peptide **19** has been treated with NaOH 1M solution following the general procedure of saponification. The desired compound **20** has been obtained in quantitative yield (50 mg); R*f* = 0.13 (EtOAc 100%). ^{1}H NMR (DMSO-d_6) δ: 9.14 (s, 1H, OH Tyr), 8.45 (1H, d, kyn NH-CO), 8.03 (m, 3H, DAla NH, Gly NH), 7.69 (d, 1H, kyn ArH), 7.23-7.14 (m, 9H, Phe ArH, kyn NH_2, kyn ArH), 6.97 (d, 2H, Tyr ArH), 6.89 (d, 1H, N*H*-Boc), 6.74 (d, 1H, kyn ArH), 6.59 (d, 2H, Tyr ArH), 4.75 (q, 1H, Phe H^α), 4.54 (m, 1H, kyn H^α), 4.28 (quint, 1H, DAla H^α), 3.95 (m, 1H, Tyr H^α), 3.65 (d, 2H, Gly H^α), 3.39 (m, 2H, Phe H^β), 3.05 (m, 2H, kyn H^β), 2.99-2.70 (m, 2H, Tyr H^β), 1.24 (s, 9H, 3*CH_3), 1.08 (d, 3H, DAla H^β).

TFA·NH_2-Tyr-DAla-Gly-Phe-Kyn-OH (**K5**): Intermediate peptide **20** has been deprotected with a mixture of TFA:DCM =1:1 at r.t. for 1h, then the solvent was evaporated in rotavapor and the residue washed with DCM, dried in rotary evaporator and high vacuum. The so obtained crude product has been purified on RP-HPLC to give final product **K5** as TFA salt in 62% overall yield; R_t = 15.14 min. ^{1}H NMR (DMSO-d_6) δ: 9.33 (s, 1H, OH Tyr), 8.54 (d, 1H, *J* = 7.5 Hz, NH-CO), 8.44 (d, 2H, *J* = 7.8 Hz, Phe NH, -COOH), 8.16 (t, 1H, Gly H^α), 8.00 (d, 1H, *J* = 8.1 Hz, DAla NH), 8.11 (brs, 3H, Tyr NH_3^+), 7.72 (d, 1H, *J* = 8.1 Hz, kyn ArH), 7.26-7.16 (m, 8H, Phe ArH, kyn NH_2, kyn ArH), 6.73 (d, 1H, *J* = 7.2 Hz, kyn ArH), 6.71 (d, 2H, *J* = 8.1 Hz, Tyr ArH), 6.67 (d, 2H, *J* = 8.2 Hz, Tyr ArH), 6.52 (t, 1H, *J* = 7.2 Hz, kyn ArH), 4.67 (q, 1H, *J* = 7.2 Hz, Phe H^α), 4.53 (m, 1H, *J* = 8.4 Hz, kyn H^α), 4.27 (quint, 1H, *J* = 7.2 Hz, DAla H^α), 3.93 (t, 1H, Tyr H^α), 3.61 (d, 2H, Gly H^α), 3.01-2.66 (m, 6H, Phe H^β, Tyr H^β, kyn

H^{β}), 1.01 (d, 3H, *J* = 6.9 Hz, DAla H^{β}). LRMS for $C_{33}H_{38}N_6O_8$ calcd. m/z = 646.2, found 646.8 [M].

Boc-Tyr-DAla-Gly-Phe-Kyn-NH_2 (**21**): Intermediate **20** was subjected to amidation following the general procedure. The desired product has been obtained in 52% yield after trituration in Et_2O (25 mg); R*f* = 0.1 (EtOAc:MeOH = 98:2). ^1H NMR (DMSO-d_6) δ: 9.14 (s, 1H, OH Tyr), 8.19-8.05 (m, 4H, -NH-CO, NH Gly, N-terminal amide kyn), 7.70 (d, 1H, kyn ArH), 7.24-7.09 (m, 9H, Phe ArH, kyn NH_2, kyn ArH, DAla NH), 6.98 (d, 3H, Tyr ArH, Phe NH), 6.85 (d, 1H, NH-Boc), 6.72 (d, 1H, kyn ArH), 6.60 (d, 2H, Tyr ArH), 6.51 (t, 1H, kyn ArH), 4.65 (q, 1H, Phe H^{α}), 4.43 (m, 1H, kyn H^{α}), 4.21 (quint, 1H, DAla H^{α}), 4.03 (m, 1H, Tyr H^{α}), 3.61 (d, 2H, Gly H^{α}), 3.00 (dd, 2H, Phe H^{β}), 2.78-2.58 (m, 4H, kyn H^{β}, Tyr H^{β}), 1.26 (s, 9H, 3*CH_3), 1.07 (d, 3H, DAla H^{β}).

TFA·NH_2-Tyr-DAla-Gly-Phe-Kyn-NH_2 (**K6**): Intermediate peptide **21** has been deprotected with a mixture of TFA:DCM =1:1 at r.t. for 1h, then the solvent was evaporated in rotavapor and the residue washed with DCM (3 times), dried in rotary evaporator and high vacuum. The so obtained crude product has been purified on RP-HPLC to give final product **K6** as TFA salt in 48% overall yield; R_t = 15.14 min. ^1H NMR (DMSO-d_6) δ: 9.34 (s, 1H, OH Tyr), 8.57 (d, 1H, *J* = 7.5 Hz, -NH-CO), 8.28 (m, 2H, Gly NH, DAla NH), 8.05 (brs, 3H, Tyr NH_3^+), 7.72 (d, 1H, *J* = 8.1 Hz, kyn ArH), 7.19 (m, 12H, Phe ArH, 2*NH_2 kyn, kyn ArH, Tyr ArH), 6.69 (d, 3H, Tyr ArH, Phe NH), 6.52 (d, 1H, *J* = 7.8 Hz, kyn ArH), 4.62 (q, 1H, *J* = 7.5 Hz, Phe H^{α}), 4.47 (m, 1H, kyn H^{α}), 4.29 (quint, 1H, DAla H^{α}), 3.94 (m, 1H, Tyr H^{α}), 3.66 (d, 2H, Gly H^{α}), 3.14-2.70 (m, 6H, Phe H^{β}, kyn H^{β}, Tyr H^{β}), 1.04 (d, 3H, *J* = 6.9 Hz, DAla H^{β}). LRMS for $C_{33}H_{39}N_7O_7$ calcd. m/z = 645.2, found 646.3 $[M+H]^+$.

RP-HPLC analytical traces of peptides KA1, K2-K6 recorded at 254 nm

KA1

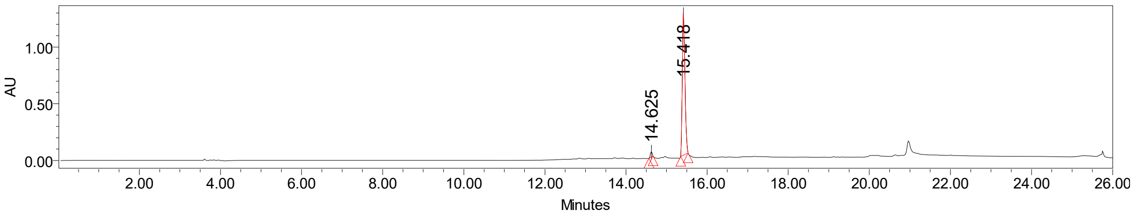

	Retention Time	Area	% Area	Height
2	15.418	5657677	97.64	1234518
1	14.625	136865	2.36	42026

K2

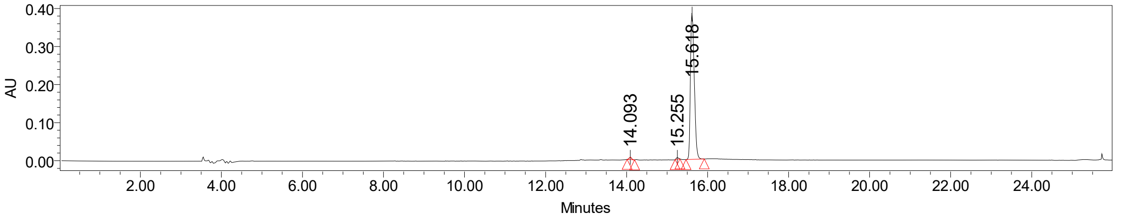

	Retention Time	Area	% Area	Height
3	15.618	2520361	98.22	384416
2	15.255	18451	0.72	4894
1	14.093	27119	1.06	6239

K3

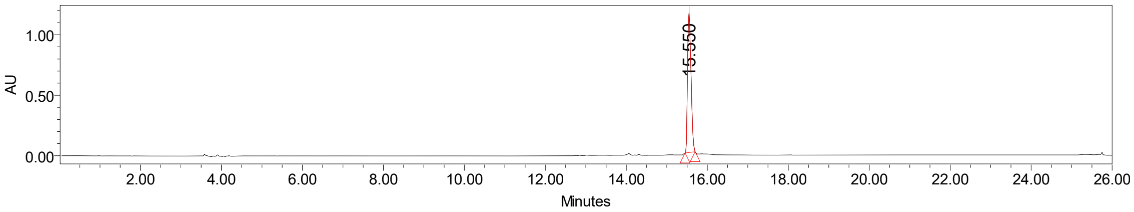

	Retention Time	Area	% Area	Height
1	15.550	7974620	100.00	1347625

K4

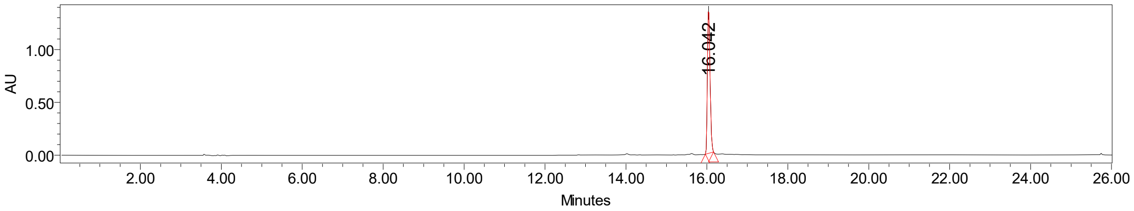

	Retention Time	Area	% Area	Height
1	16.045	5544442	100.00	1199377

K5

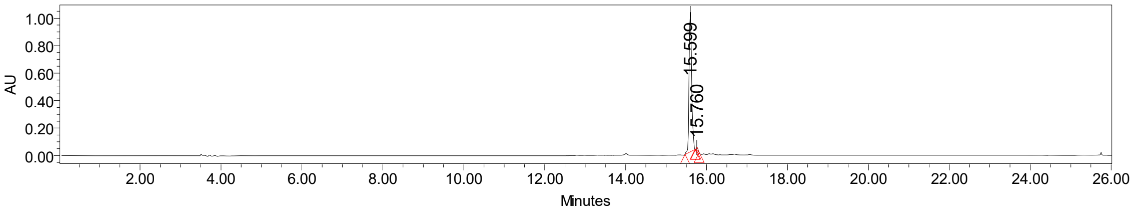

	Retention Time	Area	% Area	Height
2	15.760	62635	1.35	19976
1	15.599	4565388	98.65	1000661

K6

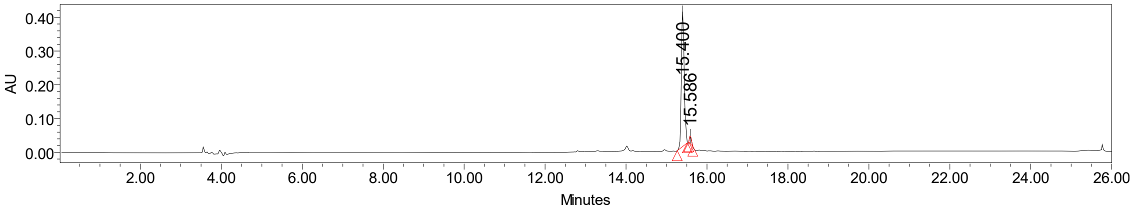

	Retention Time	Area	% Area	Height
2	15.586	73916	3.62	21019
1	15.400	1965180	96.38	395021

LOW RESOLUTION MASS SPECTROSCOPY

Peptide KA1

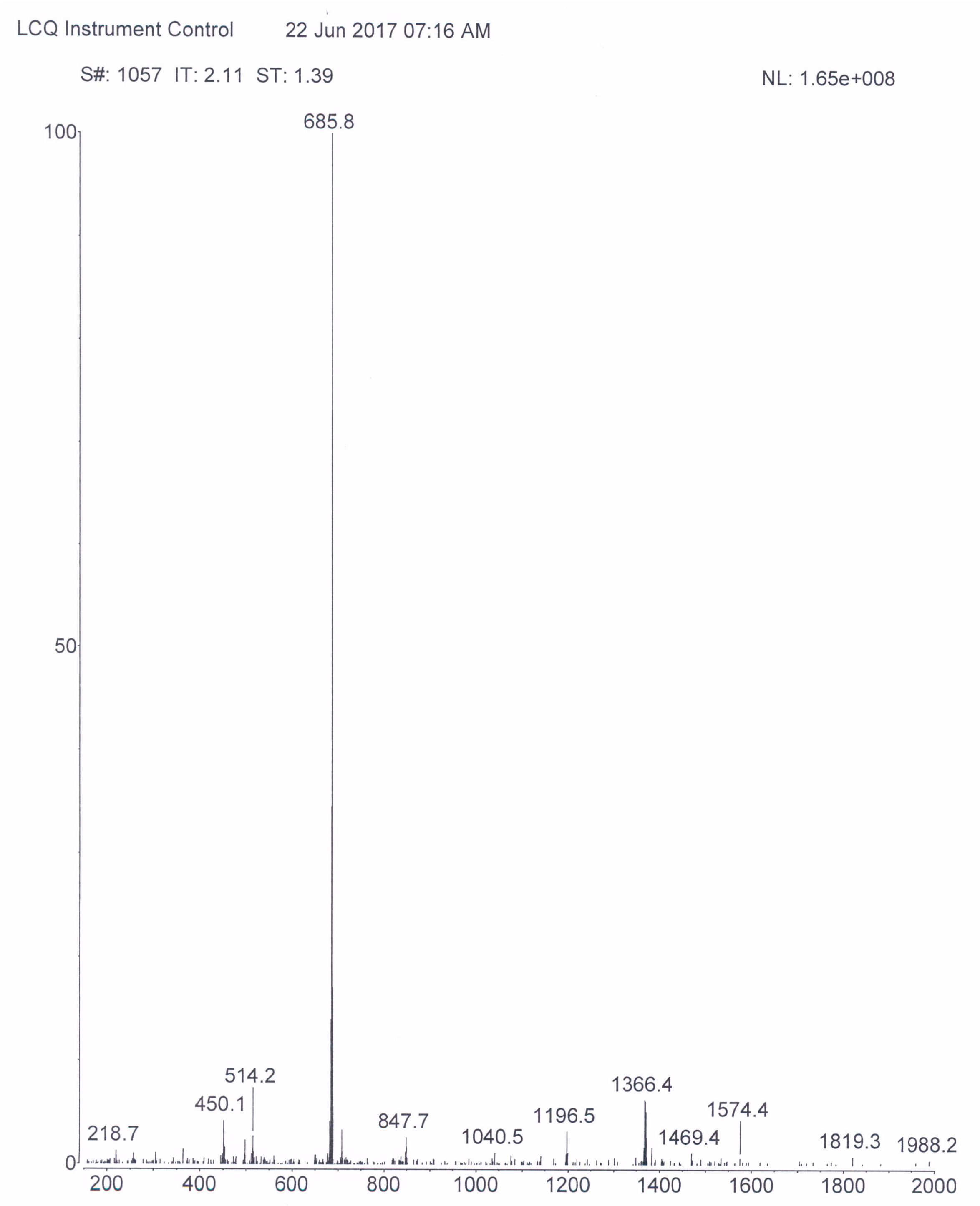

Peptide K2

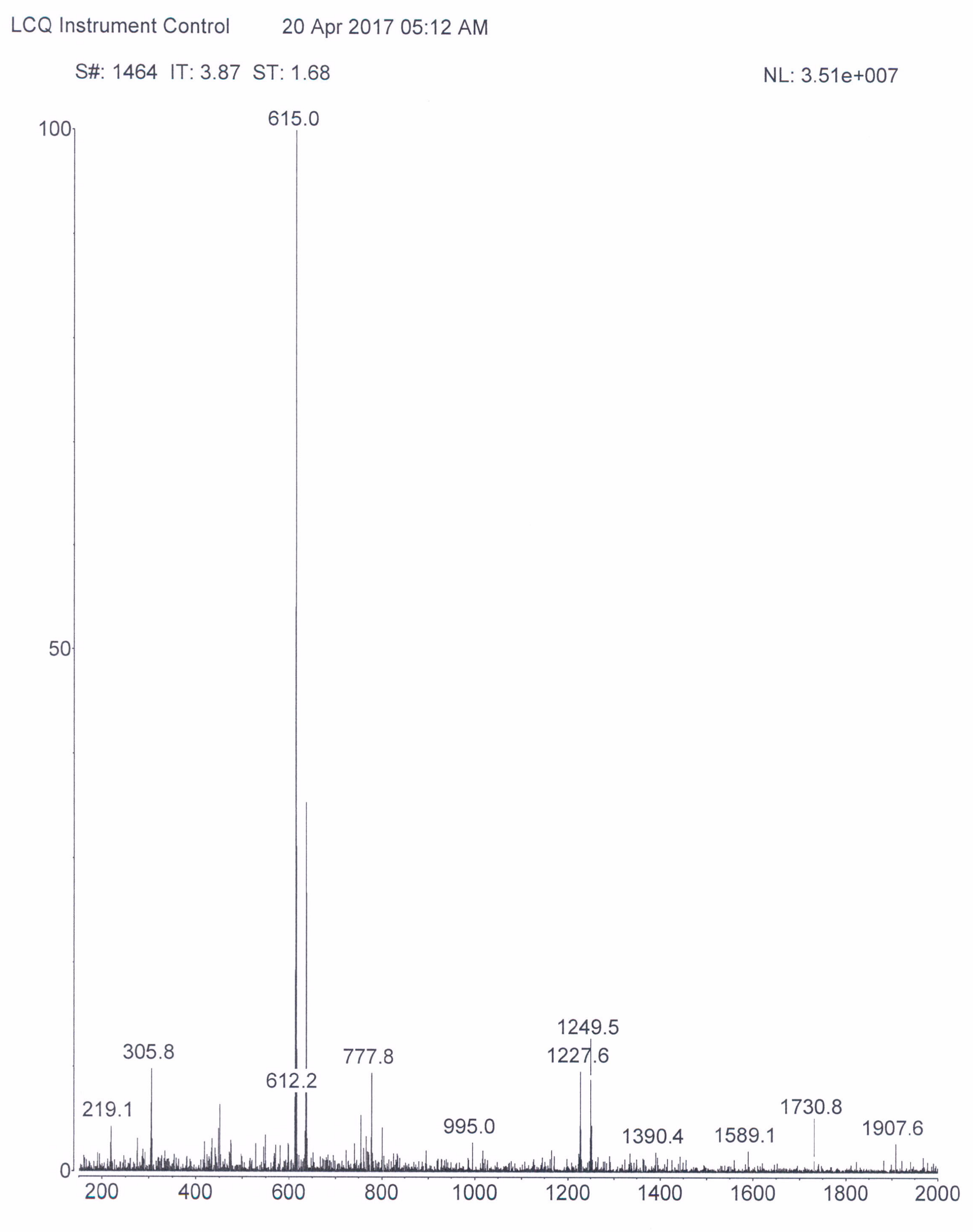

LCQ Instrument Control
20 Apr 2017 05:12 AM
S#: 1464 IT: 3.87 ST: 1.68
NL: 3.51e+007
100
50
0
615.0
305.8
219.1
612.2
777.8
995.0
1249.5
1227.6
1390.4
1589.1
1730.8
1907.6
200
400
600
800
1000
1200
1400
1600
1800
2000

Peptide K3

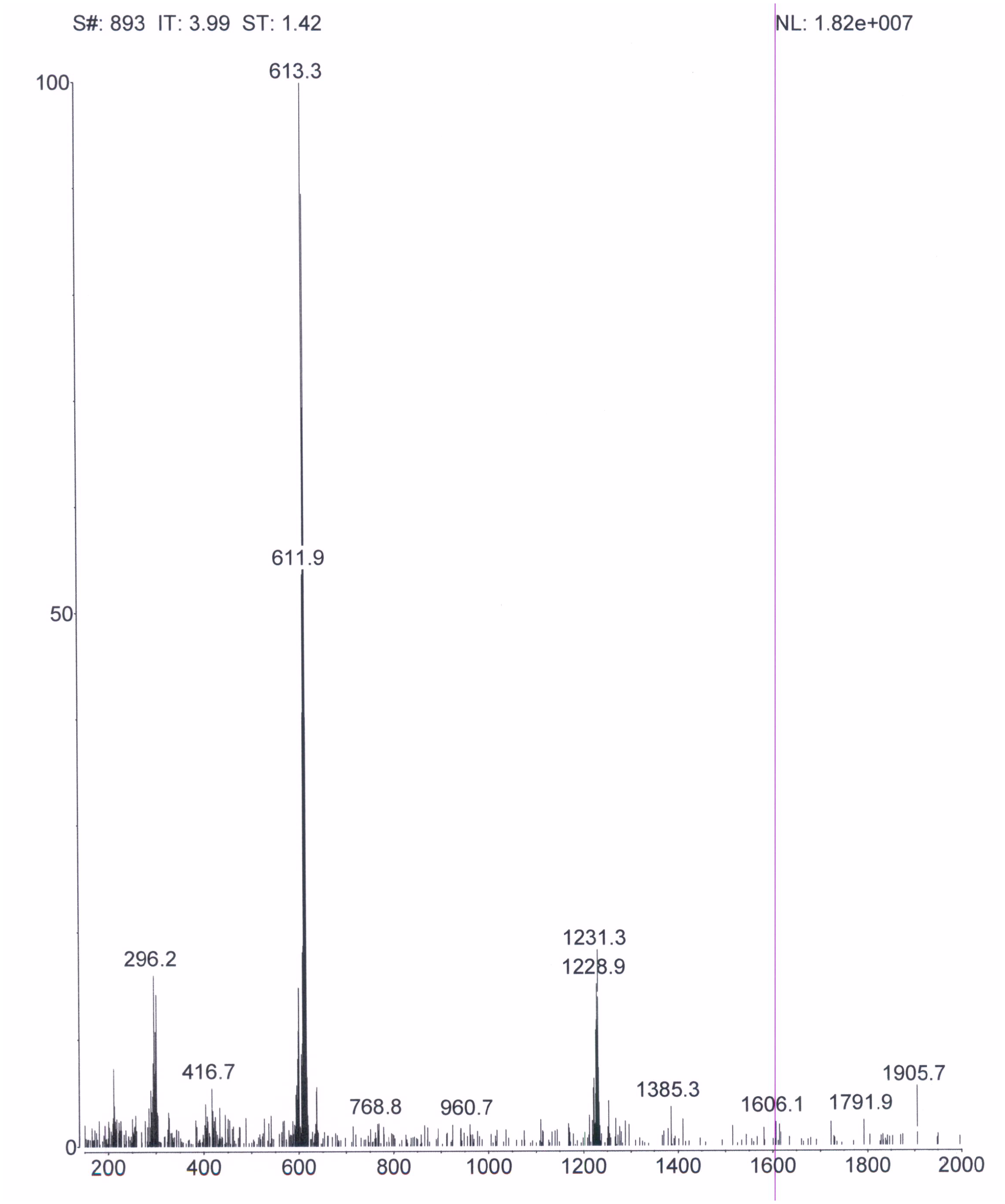
S#: 893 IT: 3.99 ST: 1.42
NL: 1.82e+007
100
50
0
613.3
611.9
296.2
416.7
768.8
960.7
1231.3
1228.9
1385.3
1606.1
1791.9
1905.7
200
400
600
800
1000
1200
1400
1600
1800
2000

Peptide K4

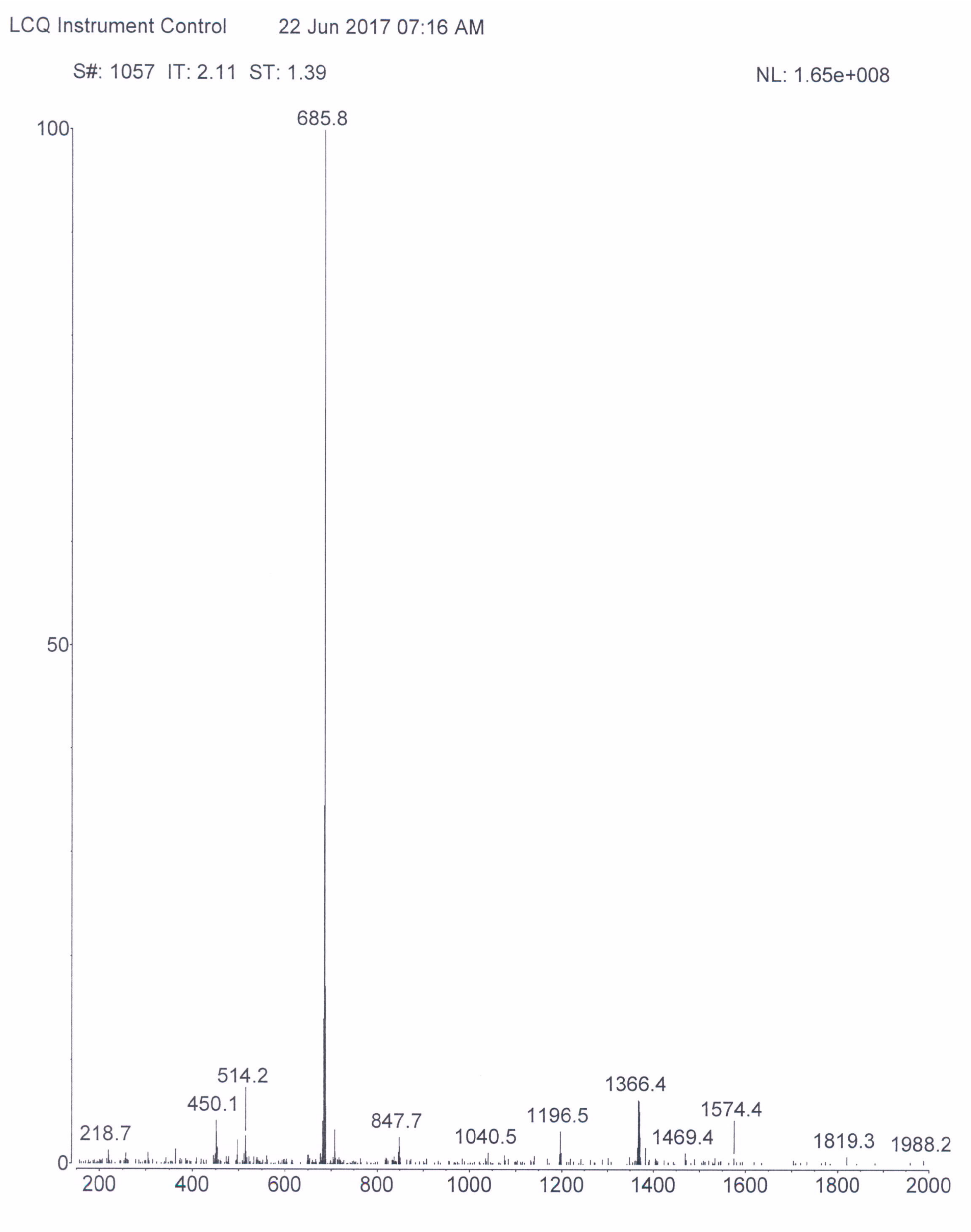

LCQ Instrument Control
22 Jun 2017 07:16 AM
S#: 1057 IT: 2.11 ST: 1.39
NL: 1.65e+008
100
50
0
685.8
514.2
450.1
218.7
847.7
1040.5
1196.5
1366.4
1469.4
1574.4
1819.3
1988.2
200
400
600
800
1000
1200
1400
1600
1800
2000

Peptide K5

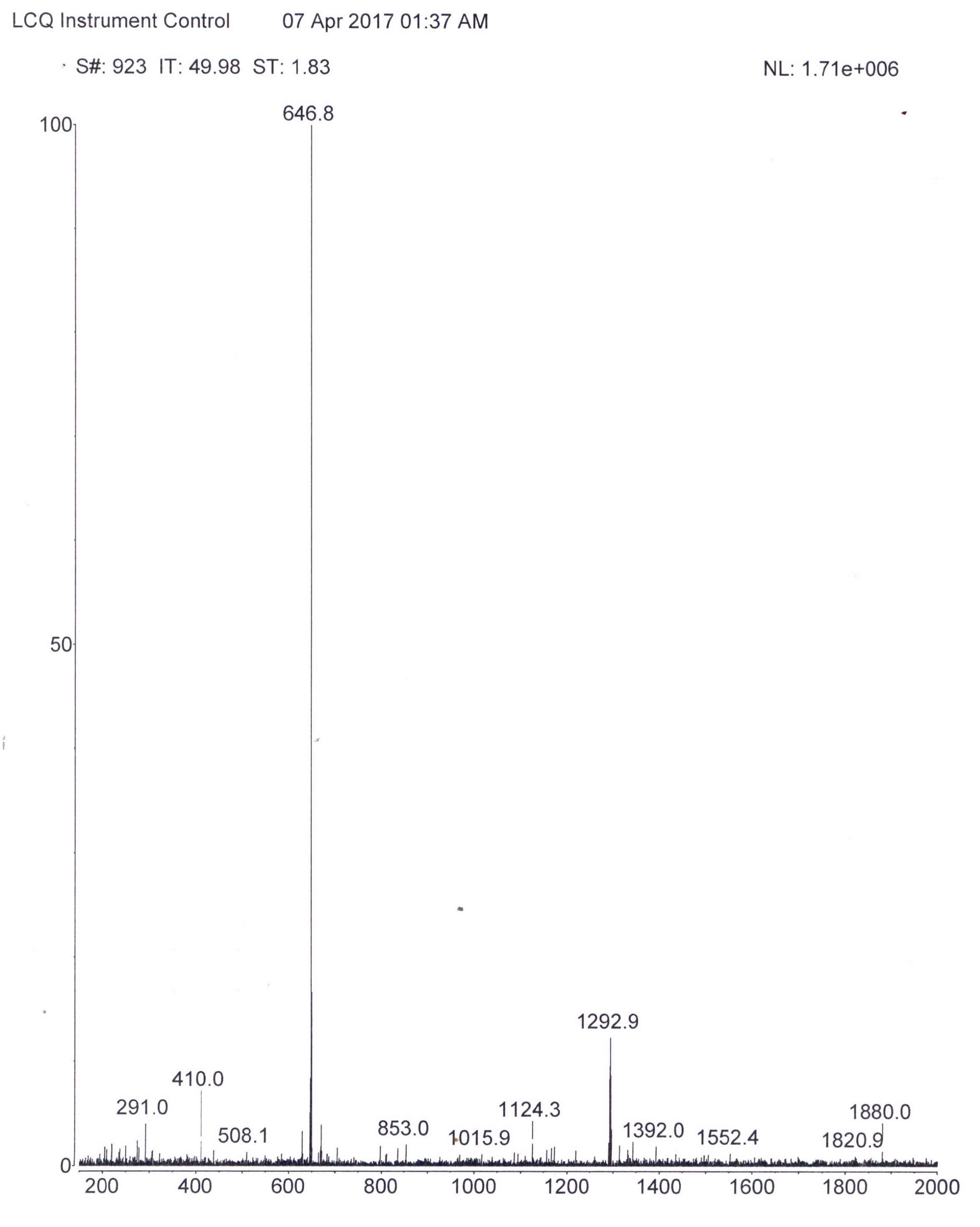
LCQ Instrument Control
07 Apr 2017 01:37 AM
S#: 923 IT: 49.98 ST: 1.83
NL: 1.71e+006
646.8
100
50
0
1292.9
410.0
291.0
508.1
853.0
1015.9
1124.3
1392.0
1552.4
1820.9
1880.0
200
400
600
800
1000
1200
1400
1600
1800
2000

Peptide K6

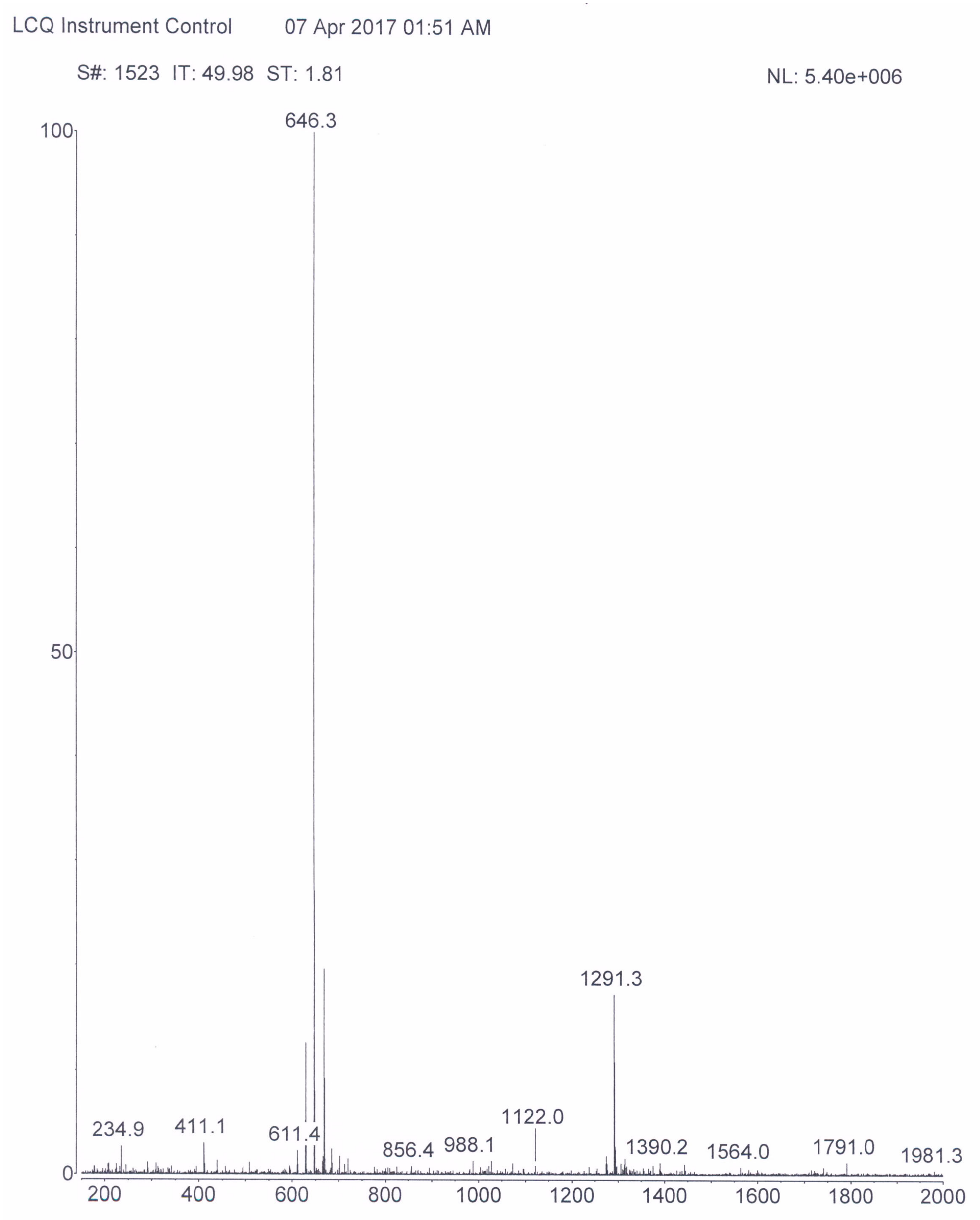
LCQ Instrument Control 07 Apr 2017 01:51 AM
S#: 1523 IT: 49.98 ST: 1.81
NL: 5.40e+006
100
50
0
646.3
1291.3
1122.0
234.9
411.1
611.4
856.4
988.1
1390.2
1564.0
1791.0
1981.3
200
400
600
800
1000
1200
1400
1600
1800
2000

^{1}H NMR KA1 in DMSO-d_6

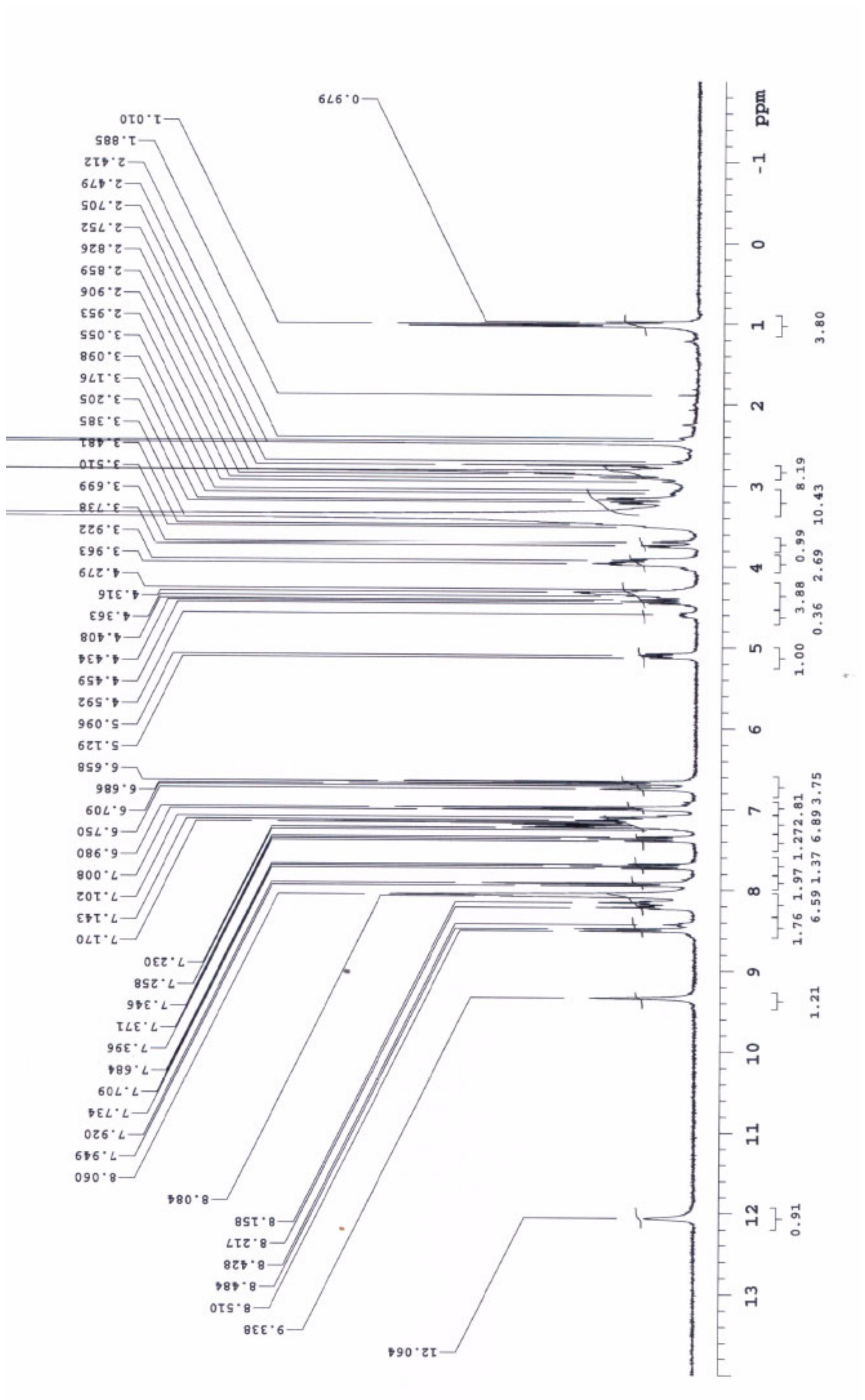

^{1}H NMR K2 in DMSO-d_6

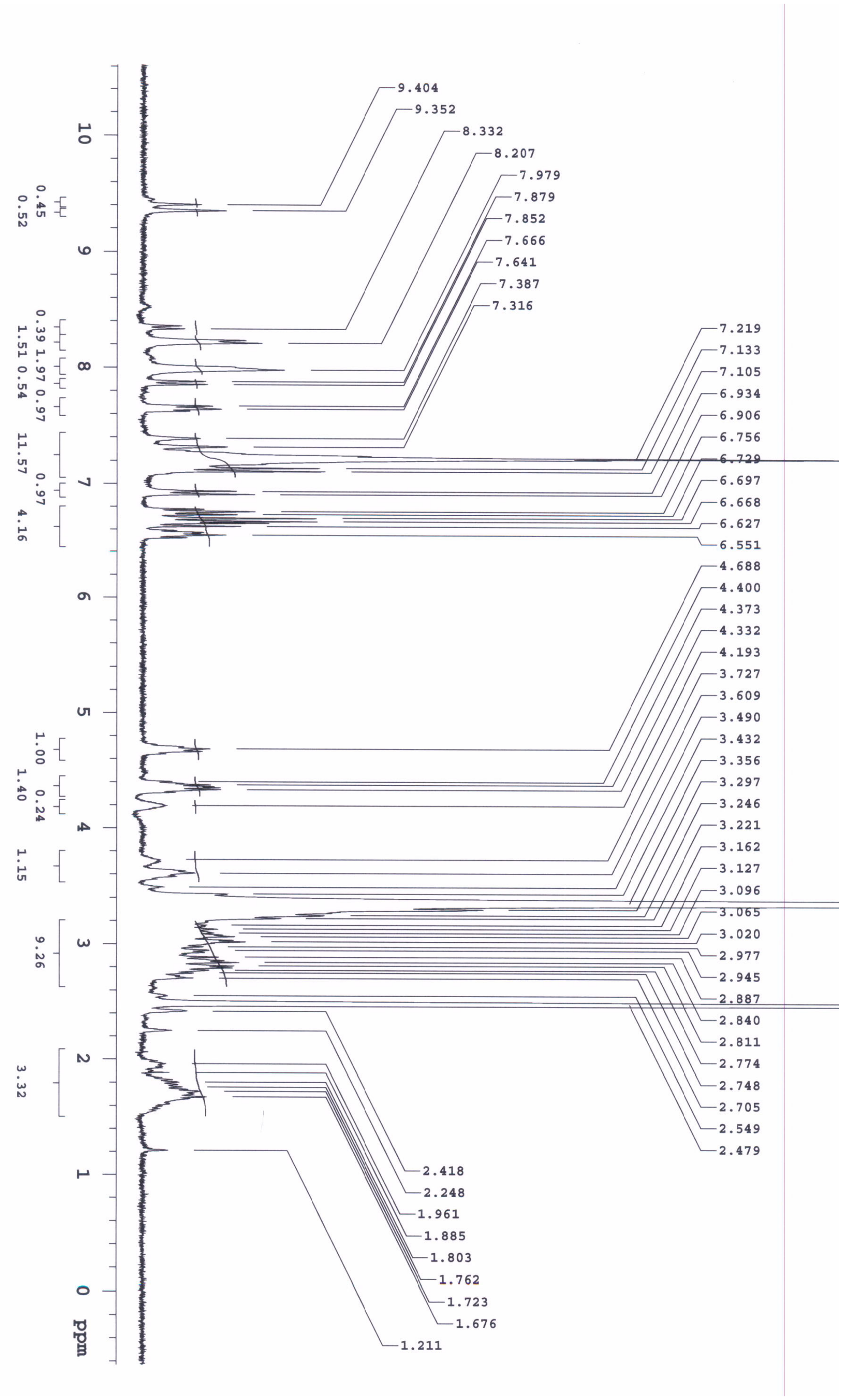

^{1}H-NMR K3 in DMSO-d$_{6}$

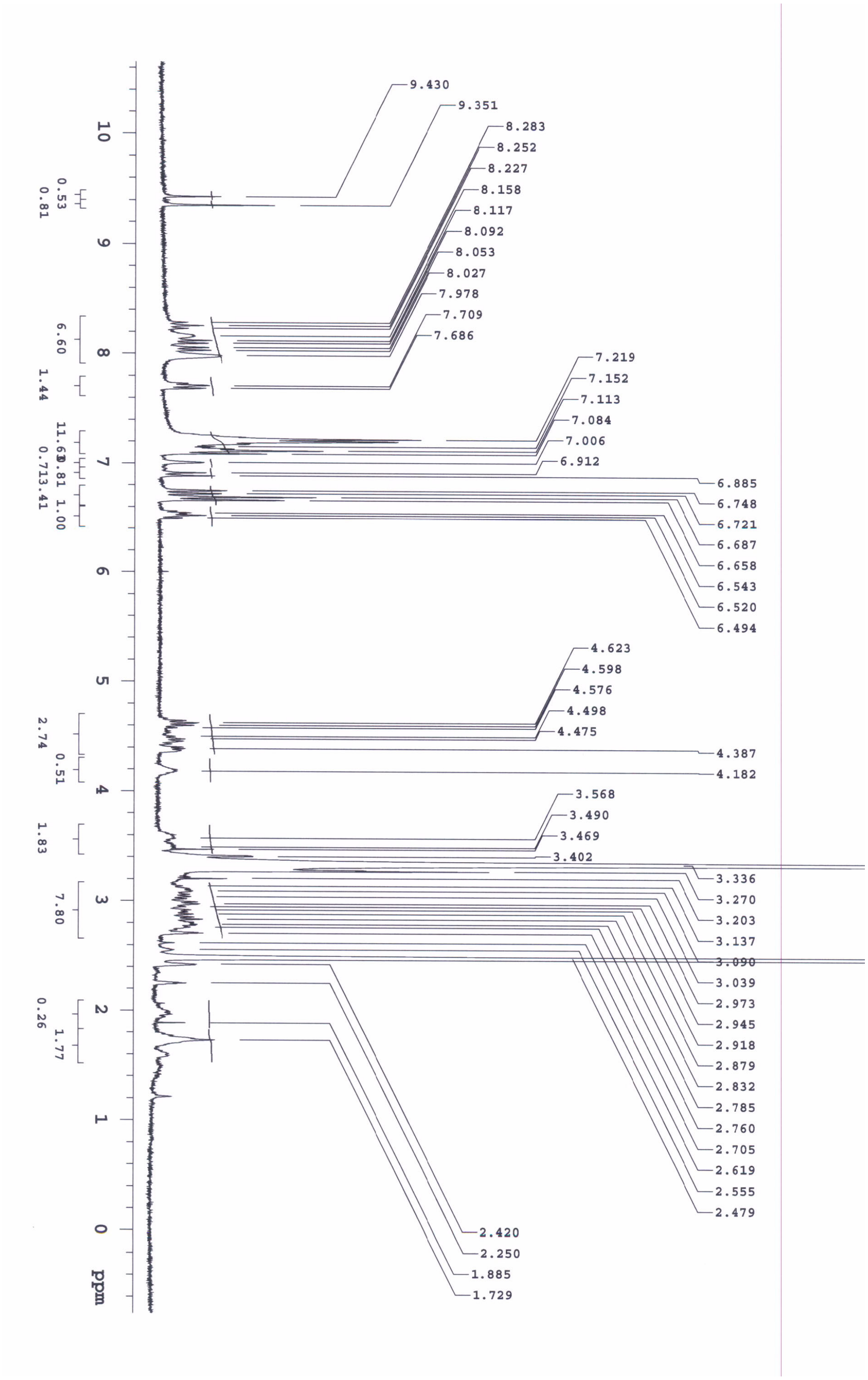

^{1}H NMR of K4 in DMSO-d_6

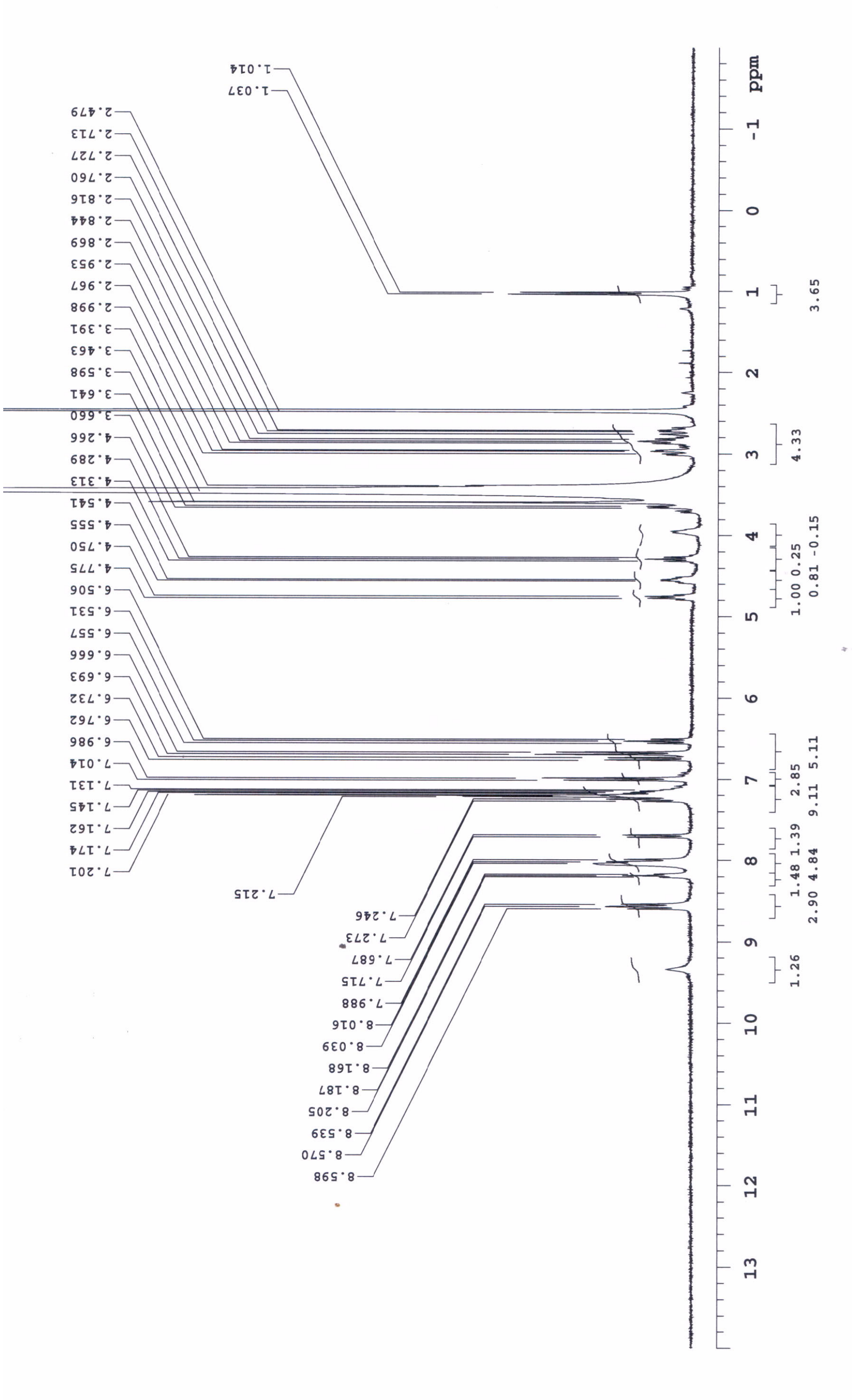

^{1}H NMR K5 in DMSO-d_6

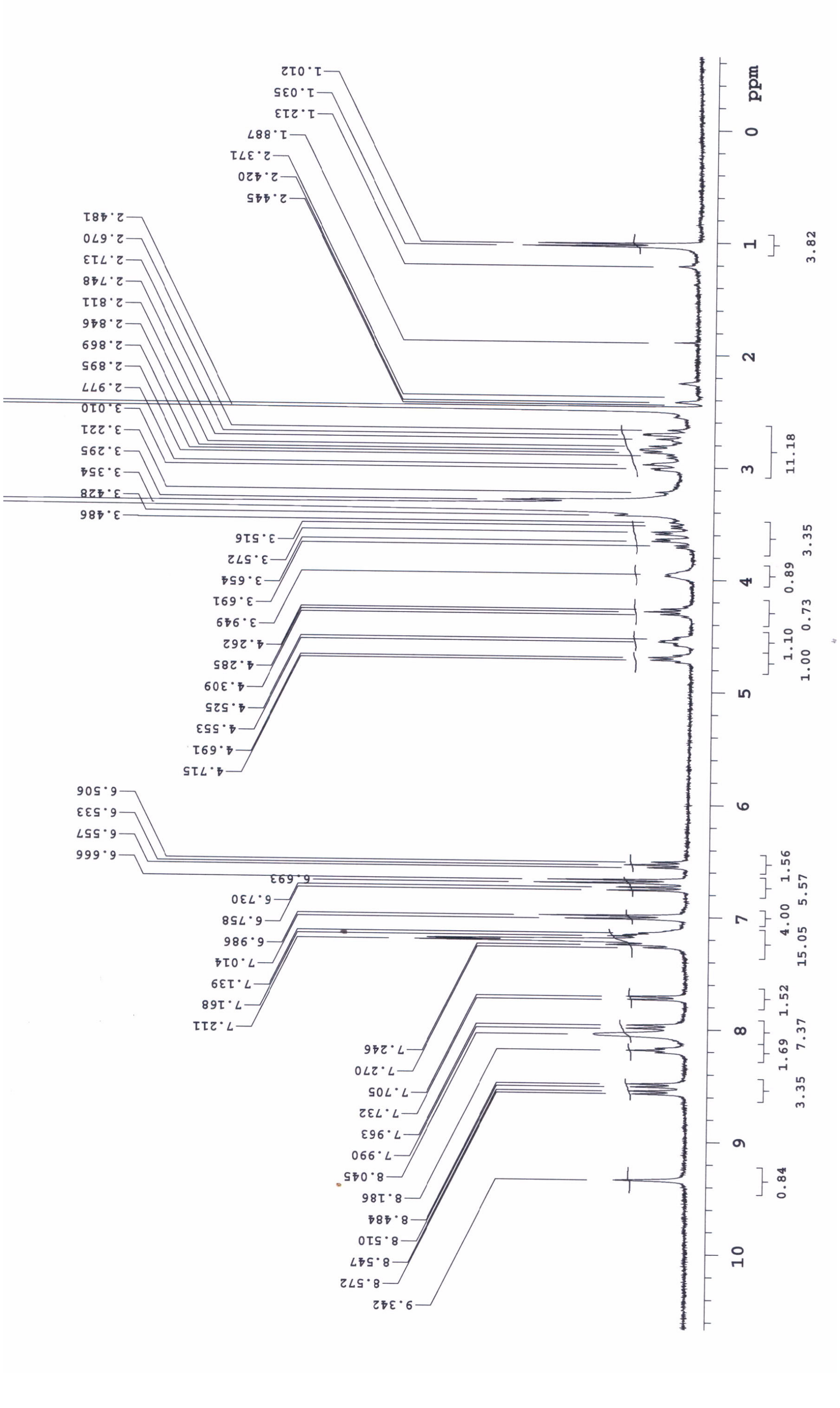

^{1}H NMR K6 in DMSO-d_6

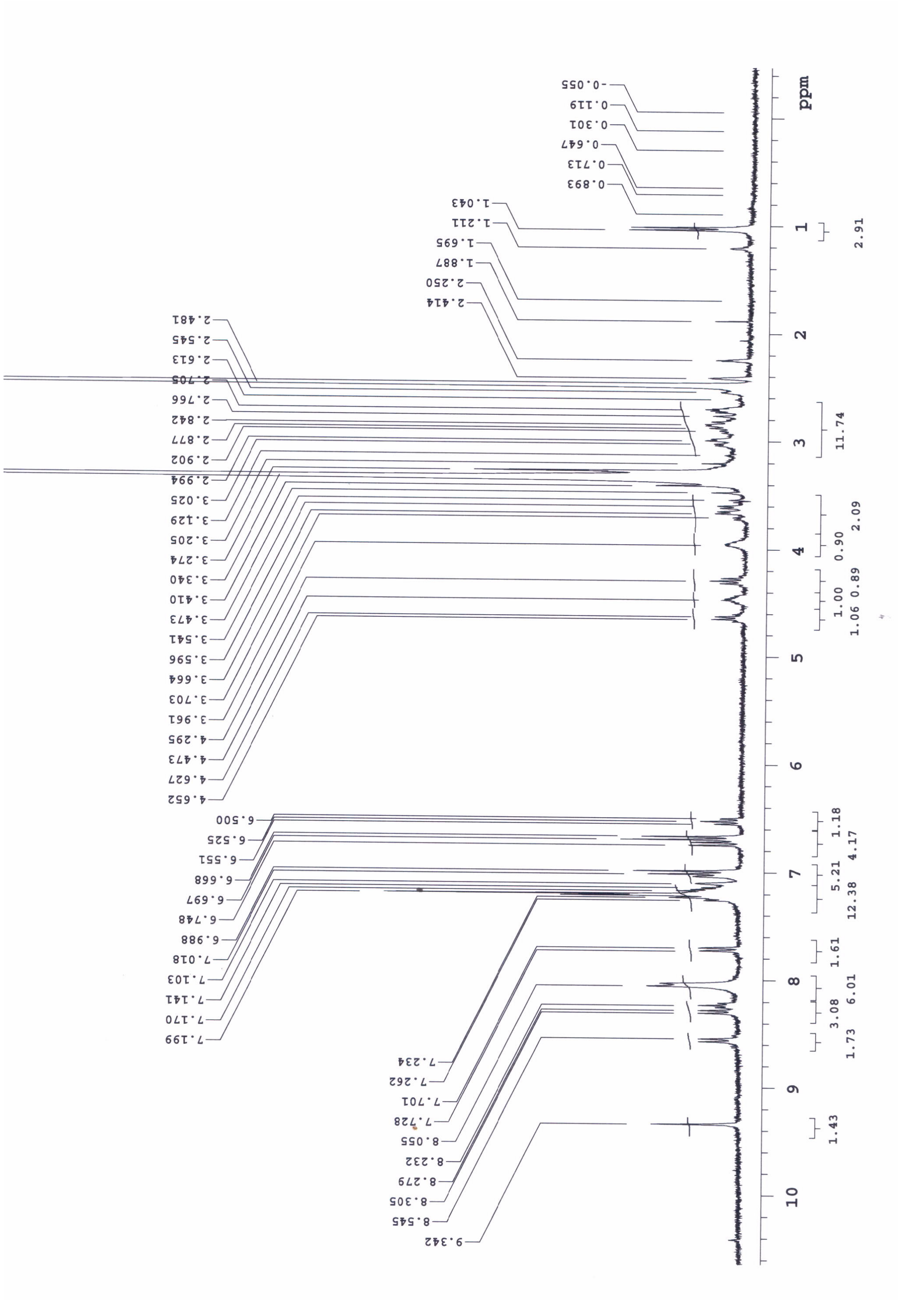

Figure S1. MOR (A), DOR (B), KOR (C) and NMDA (D) binding affinity of the novel oligopeptides.

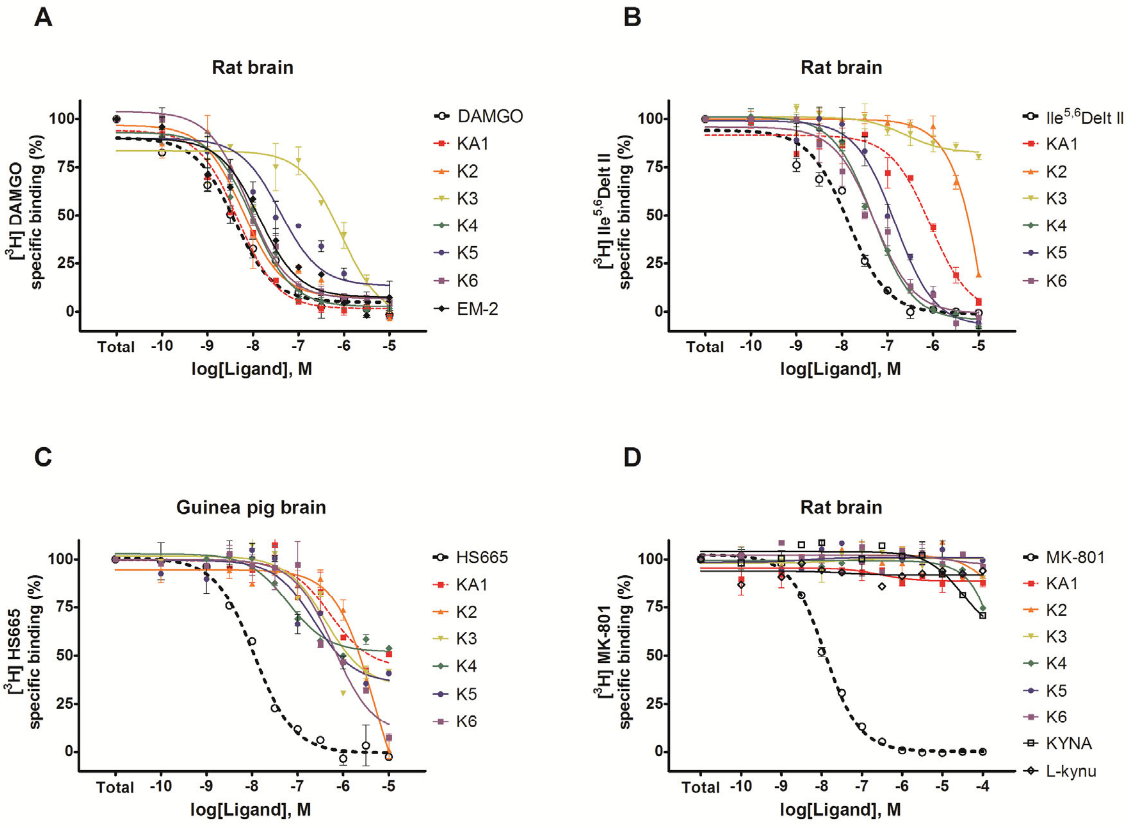

Figure legend: MOR (A) and DOR (B) binding affinity of novel oligopeptides compared to DAMGO, Ile^{5,6}Delt II, HS665 (C) and MK-801 (D), respectively in [^{3}H]DAMGO, [^{3}H]Ile^{5,6}Delt II, [^{3}H]HS665 and [^{3}H]MK-801 competition binding assays in rat and guinea pig brain membrane homogenates. Membranes were incubated with 2 nM [^{3}H]DAMGO, 2 nM [^{3}H]Ile^{5,6}Delt II, 2 nM [^{3}H]HS665 and 5 nM [^{3}H]MK-801. Values represent mean values $\pm$ S.E.M. for at least three experiments performed in duplicate.

Figure S2. The effect of oligopeptides on G-protein activity compared to DAMGO in [^{35}S]GTPγS binding assay in rat brain membrane homogenates. Figure S2 represents the specific binding of [^{35}S]GTPγS in percentage in presence of increasing concentrations (10^{-10} - 10^{-5} M) of the indicated ligands. "Total" on the x-axis indicates the basal activity of the monitored G-protein, which is measured in the absence of the ligands and represents the total specific binding of [^{35}S]GTPγS. The level of basal activity was defined as 100% and it is presented with a dotted line. Points represent means ± S.E.M. for at least three experiments performed in triplicate.

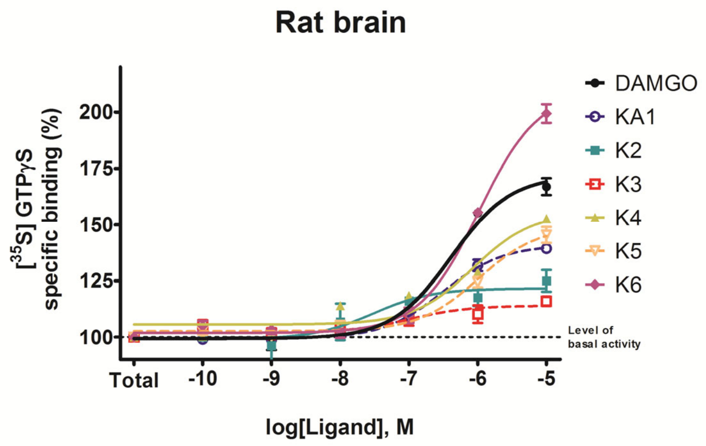

Figure S3. The effect of novel oligopeptides on G-protein activity in [^{35}S]GTPγS binding assays in the absence or presence of the selective MOR antagonist cyprodime (Cyp) and the selective DOR antagonist naltrindole (NTI) in rat brain membrane homogenates (Figure A) and the selective KOR antagonist norbinaltorphine (nor-BNI) in guinea pig brain membrane homogenates (Figure B). The level of basal activity was defined as 100% and it is presented with a dotted line. Points represent means ± S.E.M. for at least three experiments performed in triplicate. * P<0.05 ** P<0.01 *** P<0.001

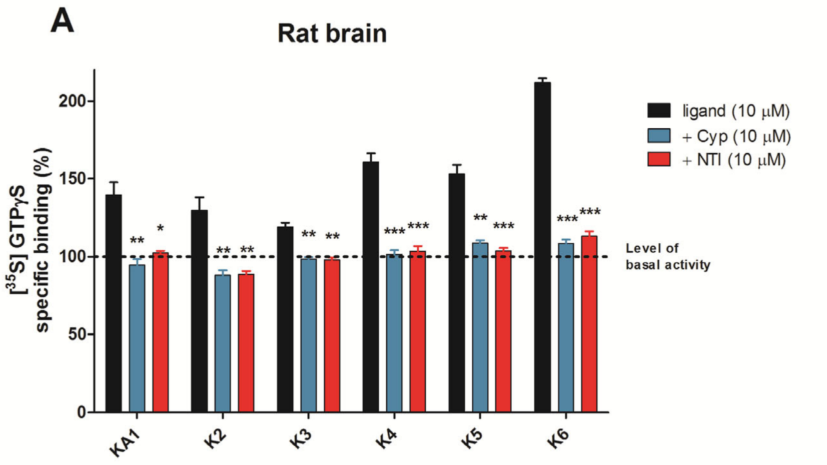

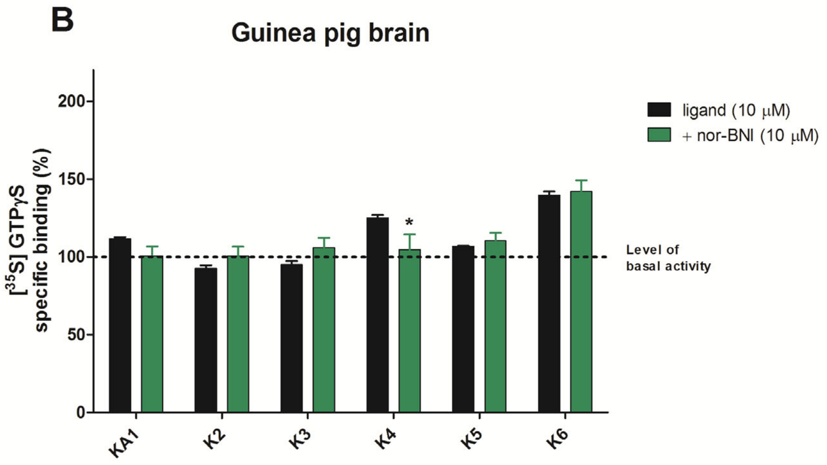

Plasma stability assay

Five calibration standards were injected into the HPLC-UV/Vis system. HPLC analyses were performed on a Waters liquid chromatograph equipped with a model 600 solvent pump and a 2996 photodiode array detector, and the mobile phase was degassed directly on-line by using Degassex, mod. DG-4400 (Phenomenex, Torrance, CA, USA). Empower v2.0 Software (Waters Spa, Milford, MA, USA) was used for data acquisition and elaboration. A reversed-phase packing column (XBridge BEH 130 C18, 4.6 × 250 mm, 5 µm; Waters Spa, Milford, MA, USA) was employed for the separation and the column was held at room temperature (22 ± 1 °C). **K6** remaining concentrations at different times are reported below. For quantitative analyses, selective detection was performed at 210 nm. Gradient elution mode was performed using a mobile phase containing a 98:1 water-ACN ratio as starting conditions and gradient program as reported.

Time (minutes)	%A (Water)	%B (ACN)
0	98.0	2.0
4	98.0	2.0
20	0.0	100.0
24	0.0	100.0
28	98.0	2.0
32	98.0	2.0

All the sample solutions were previously centrifuged and 20 µL of the supernatant was injected into the HPLC-UV/Vis system. Calibration curves from 2 to 10 µg/mL were calculated by analyzing five non-zero concentration standards prepared in freshly spiked plasma solution in triplicate and extracted. All quantitative analyses were performed at 210 nm. Calibration curves were linear with r^2 values always greater than 0.988 (n = 3). In reported chromatographic conditions, the retention times for **K6** was 16.45 minutes (± 0.4, n = 20), the substance identity was confirmed by LS-MS analysis.

Time (minutes)	**K6** Concentration (µg/mL)	**K6**% of degradation
0	10	0
5	9.325	7
10	8.588	15
30	6.435	36
60	4.443	56

References

1. Ghilardi, A.; Pezzoli, D.; Bellucci, M.C.; Malloggi, C.; Negri, A.; Sganappa, A.; Tedeschi, G.; Candiani, G.; Volonterio, A. Synthesis of multifunctional PAMAM-Aminoglycoside conjugates with enhanced transfection efficiency. *Bioconjug Chem.* **2013**, *24,* 1928-1936.

2. Tsentalovich, Y.P.; Yanshole, V.V.; Polienko, Y.F.; Morozov, S.V.; Grigor'ev, I.A. Deactivation of excited states of kynurenine covalently linked to nitroxides. *Photochem. Photobiol.* **2011**, *87,* 22-31.

3. Stefanucci, A.; Novellino, E.; Mirzaie, S.; Macedonio, G.; Pieretti, S.; Minosi, P.; Szűcs, E.; Erdei, A.I.; Zádor, F.; Benyhe, S.; Mollica, A. Opioid receptor activity and analgesic potency of DPDPE peptide analogues containing a xylene bridge. *ACS Med. Chem. Lett.* **2017**, *8,* 449-454.
